# Trustworthy AI-Powered Intrusion Detection for the Internet of Medical Things (IoMT): A Review

**DOI:** 10.3390/s26165182

**Published:** 2026-08-16

**Authors:** Jahidul Islam, Dristi Datta, Fowzia Akhter

**Affiliations:** Department of Information Technology and Engineering (ITE), Sydney Metropolitan Institute of Technology (SydneyMet), Sydney, NSW 2000, Australia; sm20241431@sydneymet.edu.au (J.I.); dristi.datta@sydneymet.edu.au (D.D.)

**Keywords:** Internet of Medical Things, Intrusion Detection Systems, Artificial Intelligence, Explainable AI, Federated Learning, Healthcare Cybersecurity, Remote Patient Monitoring, Healthcare 5.0, Cyber-Physical Systems

## Abstract

The Internet of Medical Things (IoMT) is transforming healthcare through continuous patient monitoring, telemedicine, cloud–edge services, and Healthcare 5.0. However, the rapid growth of interconnected medical devices has expanded the healthcare cyberattack surface, making intelligent intrusion detection essential for protecting sensitive medical data and ensuring resilient clinical operations. Existing reviews examine specific aspects of AI-powered intrusion detection but rarely provide a deployment-oriented synthesis linking technical performance with operational and clinical requirements. This review critically examines Artificial Intelligence (AI)-powered Intrusion Detection Systems (IDSs) for IoMT across six analytical dimensions: detection performance, explainability, privacy preservation, computational efficiency, benchmarking practices, and cross-dataset generalization. This structured narrative review adopted the PRISMA 2020 framework to ensure transparent record identification, screening, and reporting, with evidence synthesized qualitatively rather than through quantitative meta-analysis. A total of 5127 records published between 2021 and 2026 were screened, resulting in 24 primary studies supported by 115 complementary studies. The findings show that machine learning, deep learning, hybrid AI, Explainable Artificial Intelligence (XAI), Federated Learning (FL), blockchain-assisted security, and edge intelligence have significantly advanced IoMT intrusion detection. However, despite benchmark accuracies often exceeding 95%, deployment remains constrained by dataset dependency, weak cross-dataset generalization, computational overhead, limited explainability, fragmented benchmarking, and insufficient operational validation. This review identifies deployment readiness, rather than predictive accuracy alone, as the principal challenge for next-generation healthcare cybersecurity and provides a practical framework for developing trustworthy, interoperable, privacy-preserving, and deployment-ready IoMT cybersecurity architectures supported by standardized evaluation protocols.

## 1. Introduction

The rapid digital transformation of healthcare has been driven by the convergence of the Internet of Medical Things (IoMT), Artificial Intelligence (AI), cloud–edge computing, and Healthcare 5.0, enabling intelligent, interconnected, and data-driven healthcare services. Through wearable sensors, implantable medical devices, Electronic Health Records (EHRs), and Remote Patient Monitoring (RPM) platforms, these technologies support continuous patient monitoring, personalized healthcare, telemedicine, and real-time clinical decision-making beyond traditional hospital settings [1,2,3,4,5,6,7,8,9,10,11,12,13,14,15,16]. However, the increasing interconnection of medical devices, communication networks, and cloud–edge infrastructures has substantially expanded the healthcare cyberattack surface, making resilient cybersecurity essential for protecting sensitive patient data, maintaining service continuity, and supporting trustworthy digital healthcare ecosystems [10,17].

Unlike conventional enterprise networks, IoMT environments combine resource-constrained medical devices, heterogeneous communication protocols, distributed healthcare infrastructures, and safety-critical clinical applications, creating cybersecurity challenges that cannot be adequately addressed using traditional security mechanisms [4,6]. Consequently, healthcare systems are increasingly exposed to ransomware, malware, denial-of-service attacks, spoofing, unauthorized access, data manipulation, and attacks targeting AI-enabled services, threatening patient privacy, operational continuity, and clinical safety [17,18,19,20,21,22,23,24,25]. These threats have accelerated the evolution of Intrusion Detection Systems (IDSs) from conventional signature-based approaches toward intelligent, adaptive, and deployment-oriented AI-powered architectures capable of identifying sophisticated and previously unseen attacks.

Recent advances in machine learning, deep learning, hybrid AI, Explainable Artificial Intelligence (XAI), Federated Learning (FL), blockchain-assisted security, and edge intelligence have substantially improved intrusion detection performance in IoMT environments [26,27,28,29,30,31,32,33,34,35]. Nevertheless, the literature consistently demonstrates that high benchmark performance—often exceeding 95% detection accuracy—does not necessarily translate into successful deployment within real healthcare environments. Persistent challenges related to cross-dataset generalization, computational efficiency, explainability, interoperability, scalability, and long-term operational robustness continue to limit practical adoption, indicating that deployment readiness has become the defining challenge for next-generation AI-powered IoMT cybersecurity [20,23,24,36,37,38,39,40,41].

Although numerous surveys have examined AI-powered intrusion detection and related IoMT security technologies, comparatively few provide an integrated assessment of the technical, operational, and deployment factors governing real-world Healthcare 5.0 adoption. Table 1 positions the present review within the existing literature.

As summarized in Table 1, existing reviews have made significant contributions to AI-powered intrusion detection, Explainable Artificial Intelligence (XAI), Federated Learning (FL), blockchain-assisted security, Healthcare 5.0, and Remote Patient Monitoring (RPM). However, most examine these topics independently or emphasize specific technologies rather than providing an integrated assessment of the factors governing real-world deployment. In particular, deployment-oriented challenges—including cross-dataset generalization, benchmarking consistency, computational efficiency, explainability, interoperability, scalability, and long-term operational validation—remain fragmented across the existing literature.

Motivated by these limitations, this review provides a comprehensive and deployment-oriented synthesis of AI-powered intrusion detection systems for the Internet of Medical Things. Unlike previous reviews that primarily focus on individual technologies, this study integrates detection performance, explainability, privacy preservation, collaborative intelligence, benchmarking practices, computational efficiency, cross-dataset generalization, and deployment readiness within a unified analytical framework. By combining technical analysis with operational and Healthcare 5.0 perspectives, the review identifies the principal barriers preventing the translation of AI-powered IDSs from laboratory research into real-world healthcare deployment and establishes strategic priorities for next-generation trustworthy, scalable, and deployment-ready IoMT cybersecurity systems.

The principal contributions of this review are summarized as follows:

1. A unified analytical framework for evaluating AI-powered IoMT intrusion detection systems across six complementary dimensions: detection performance, explainability, privacy preservation, computational efficiency, benchmarking practices, and cross-dataset generalization.
2. A deployment-oriented synthesis integrating Explainable Artificial Intelligence (XAI), Federated Learning (FL), blockchain-assisted security, edge intelligence, Healthcare 5.0, and Remote Patient Monitoring (RPM) to assess trustworthy and scalable healthcare cybersecurity.
3. A critical analysis of 24 primary studies identifying the principal barriers to real-world deployment, including dataset dependency, limited cross-dataset validation, inconsistent benchmarking, insufficient explainability, and inadequate deployment-oriented evaluation.
4. A transparent and reproducible PRISMA 2020-guided review comprising 24 primary studies supported by 115 complementary studies that provide conceptual, methodological, and technological context.
5. A future research roadmap outlining priorities for trustworthy, explainable, privacy-preserving, standardized, and deployment-ready AI-powered IoMT intrusion detection systems.

Accordingly, this review shifts the focus from accuracy-centred IDS comparison toward trustworthy, deployment-ready IoMT cybersecurity.

The remainder of this paper is organized as follows. Section 2 presents the review methodology. Section 3 synthesizes the characteristics and findings of the included studies. Section 4 discusses the principal insights and their implications for deployment-ready IoMT cybersecurity. Section 5 outlines future research directions, and Section 6 concludes the paper.

## 2. Review Methodology

### 2.1. Search and Selection Strategy

This structured narrative review was guided by the Preferred Reporting Items for Reviews and Meta-Analyses (PRISMA 2020) framework to support transparent identification, screening, selection, and reporting of literature investigating Artificial Intelligence (AI)-powered Intrusion Detection Systems (IDSs) for the Internet of Medical Things (IoMT) [42]. PRISMA 2020 was used as a reporting and transparency framework; the evidence was synthesized through structured qualitative comparison rather than quantitative meta-analysis. The review synthesized the literature across six analytical dimensions: detection performance, explainability, privacy preservation, computational efficiency, benchmarking practices, and cross-dataset generalization. In addition, deployment-oriented considerations relevant to Remote Patient Monitoring (RPM) and Healthcare 5.0 environments were incorporated to evaluate the practical applicability of AI-powered IDSs beyond benchmark performance.

A comprehensive literature search was conducted across eight electronic databases: IEEE Xplore, Google Scholar, Scopus, Web of Science, ACM Digital Library, ScienceDirect, SpringerLink, and PubMed. These databases were selected to provide complementary coverage of engineering, cybersecurity, artificial intelligence, networking, healthcare technologies, digital health, and biomedical research. The search covered studies published between 2021 and 2026 using a common Boolean search strategy with minor syntax adaptations for individual database interfaces. Database-specific search dates, filters, search methods, and retrieval statistics are summarized in Table 2.

The search strategy combined keywords related to IoMT, intrusion detection, artificial intelligence, machine learning, deep learning, explainable AI, federated learning, blockchain, healthcare cybersecurity, medical device security, and Remote Patient Monitoring. The core Boolean search string was as follows:

(“Internet of Medical Things” OR “IoMT”) AND (“Intrusion Detection System” OR “IDS” OR “Anomaly Detection”) AND (“Artificial Intelligence” OR “Machine Learning” OR “Deep Learning” OR “Explainable Artificial Intelligence” OR “Explainable AI” OR “XAI” OR “Federated Learning” OR “Blockchain”) AND (“Healthcare Cybersecurity” OR “Medical Device Security” OR “Remote Patient Monitoring” OR “RPM” OR “Healthcare 5.0”).

Because Google Scholar returns relevance-ranked results and indexes records of varying quality, searches were conducted using publication-year intervals (2021–2022, 2022–2023, 2023–2024, 2024–2025, and 2025–2026). This process retrieved 798, 810, 817, 813, and 807 records, respectively, yielding 4045 records in total. Approximately 800 records were screened within each interval according to title relevance, abstract relevance, keyword alignment, source quality, and relevance to AI-powered IoMT intrusion detection before duplicate removal and eligibility assessment.

All retrieved records were imported into Zotero for reference management, duplicate detection, and screening. Title and abstract screening was initially performed by the first author, whereas full-text eligibility assessment and methodological quality evaluation were independently verified by the co-authors. Studies with uncertain eligibility were resolved through discussion using the predefined inclusion criteria, methodological quality, relevance to AI-powered IoMT intrusion detection, and contribution to the analytical objectives of the review.

**Figure 1:**
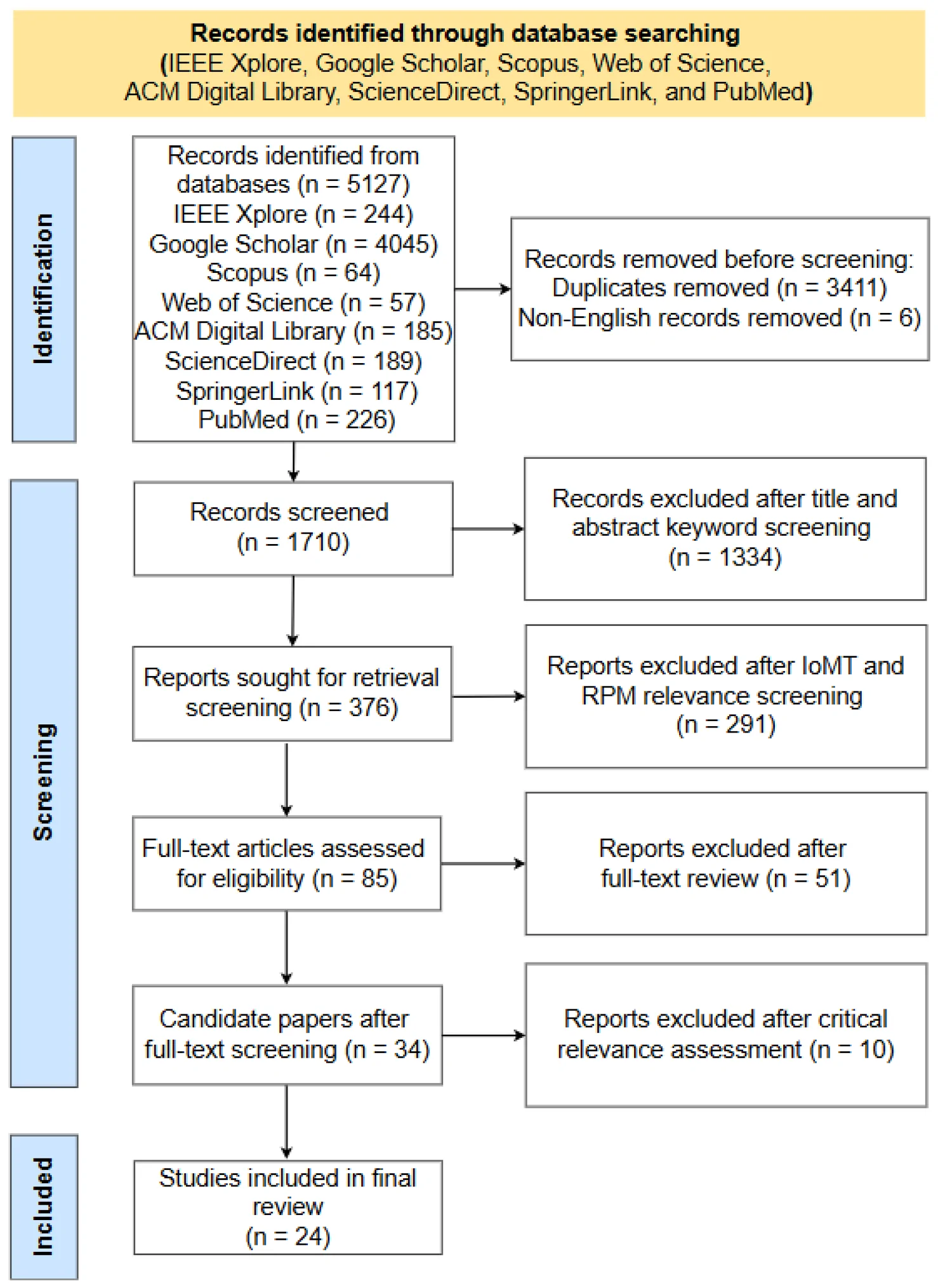
PRISMA-guided study selection process illustrating identification, duplicate removal, language filtering, title screening, abstract screening, full-text eligibility assessment, quality assessment, and final study inclusion.

The literature search identified 5127 records before screening. Following duplicate removal, language filtering, title and abstract screening, full-text eligibility assessment, and methodological quality evaluation, 24 studies satisfied all predefined eligibility criteria and were retained as the primary evidence base for formal comparative synthesis. The complete PRISMA-guided study selection process is presented in Figure 1.

The screening process comprised four sequential stages. First, duplicate records identified across the eight databases were removed using Zotero’s duplicate detection function. Second, title and abstract screening excluded studies outside the scope of AI-powered IoMT intrusion detection. Third, potentially eligible studies underwent full-text assessment against the predefined eligibility criteria. Finally, the remaining studies were evaluated using a structured quality assessment framework to determine their suitability for formal comparative synthesis.

Although the final bibliography contains 139 cited references, only 24 studies satisfied all predefined eligibility and methodological quality criteria and therefore constituted the primary evidence base for the review. The remaining 115 studies were incorporated as supporting literature to provide theoretical background, methodological justification, Healthcare 5.0 perspectives, Remote Patient Monitoring (RPM) context, and discussion of emerging technologies. These supporting studies were intentionally excluded from the formal comparative analysis because their role was to contextualize and strengthen the interpretation of the primary evidence rather than contribute directly to the structured synthesis.

Study selection was guided by predefined inclusion and exclusion criteria to ensure methodological consistency, transparency, reproducibility, and direct relevance to AI-powered IoMT intrusion detection. The detailed database search strategy is summarized in Table 2, while the predefined eligibility criteria are presented in Table 3.

### 2.2. Quality Assessment

A structured methodological quality assessment was conducted to ensure that the primary evidence included in this review was scientifically reliable, methodologically transparent, and directly relevant to AI-powered intrusion detection for the Internet of Medical Things (IoMT). Quality assessment was performed after the PRISMA-guided full-text eligibility screening of the 34 candidate studies and served as the final stage for determining inclusion in the comparative synthesis.

Each study was evaluated using five predefined quality criteria: (i) relevance to IoMT intrusion detection and healthcare cybersecurity, (ii) methodological transparency and reproducibility, (iii) adequacy of dataset description and evaluation reporting, (iv) experimental or comparative validation, and (v) contribution to explainability, privacy preservation, deployment readiness, or broader healthcare cybersecurity. Each criterion was scored on a three-point scale (0–2), where 0 indicated insufficient evidence, 1 indicated partial fulfilment, and 2 indicated strong fulfilment, resulting in a maximum quality score of 10.

Studies scoring below 5 were excluded because they demonstrated insufficient methodological rigor, limited technical relevance, or inadequate experimental reporting. Studies achieving a score of 5 or above were retained for the primary comparative synthesis. This threshold ensured that the evidence base maintained methodological quality while preserving representative coverage of experimental studies, reviews, and framework papers within the multidisciplinary IoMT cybersecurity domain.

Table 4 summarizes the overall quality assessment outcomes. Of the 34 full-text candidate studies, 24 achieved the predefined inclusion threshold (score ≥ 5) and were retained as the primary evidence base, whereas 10 studies were excluded because they did not satisfy the minimum quality requirements. The detailed study-level quality assessment for the 24 included studies is presented in Table 5.

The predominance of higher quality scores among the included studies should be interpreted in the context of the sequential study selection process rather than as evidence of limited discriminating power of the quality assessment framework. Methodological quality assessment was conducted only after the 34 candidate studies had successfully passed the PRISMA-guided title screening, abstract screening, and full-text eligibility assessment. Consequently, the remaining candidate pool already consisted of studies that satisfied the predefined relevance and eligibility criteria before formal methodological quality assessment was undertaken. The primary purpose of the quality assessment was therefore to determine whether each eligible study met the minimum methodological standard required for inclusion in the comparative synthesis rather than to generate a broad distribution of quality scores across the entire literature. Accordingly, 10 of the 34 eligible studies (29.4%) did not satisfy the predefined inclusion criterion and were excluded, whereas the remaining 24 studies demonstrated generally strong methodological quality, resulting in the concentration of higher scores presented in Table 5.

### 2.3. Characteristics of Included Studies

Following quality assessment, 24 primary studies were retained as the evidence base for comparative analysis and thematic synthesis. Because the objective of this review extends beyond algorithmic IDS performance to deployment readiness, the evidence base intentionally includes both direct IoMT intrusion detection studies and closely related deployment-enabling studies, including federated learning, blockchain-assisted trust, explainable AI, Remote Patient Monitoring (RPM) security, Healthcare 5.0, and broader IoMT cybersecurity frameworks. Because many studies address multiple challenges simultaneously, thematic categories were treated as overlapping rather than mutually exclusive.

Table 6 summarizes the thematic distribution of the included studies. The evidence shows that AI-powered intrusion detection remains the dominant research focus, while federated learning, blockchain-assisted trust, explainability, Healthcare 5.0, and RPM security are increasingly integrated into broader deployment-oriented IoMT cybersecurity frameworks.

The thematic classification provides a high-level overview of the evidence base, while Table 7 presents the detailed study-level extraction, including study type, datasets, AI/IDS methods, explainability mechanisms, privacy-preserving technologies, evaluation metrics, principal findings, limitations, and quality scores.

Table 7 demonstrates substantial methodological diversity across the 24 primary studies. Traditional machine learning remains widely used, but recent studies increasingly employ deep learning, federated learning, blockchain-assisted security, edge intelligence, and explainable AI. Despite strong reported detection performance, recurring limitations include limited cross-dataset validation, dependence on a small number of benchmark datasets, weak real-world healthcare evaluation, inconsistent computational-efficiency reporting, and limited explainability.

**Figure 2:**
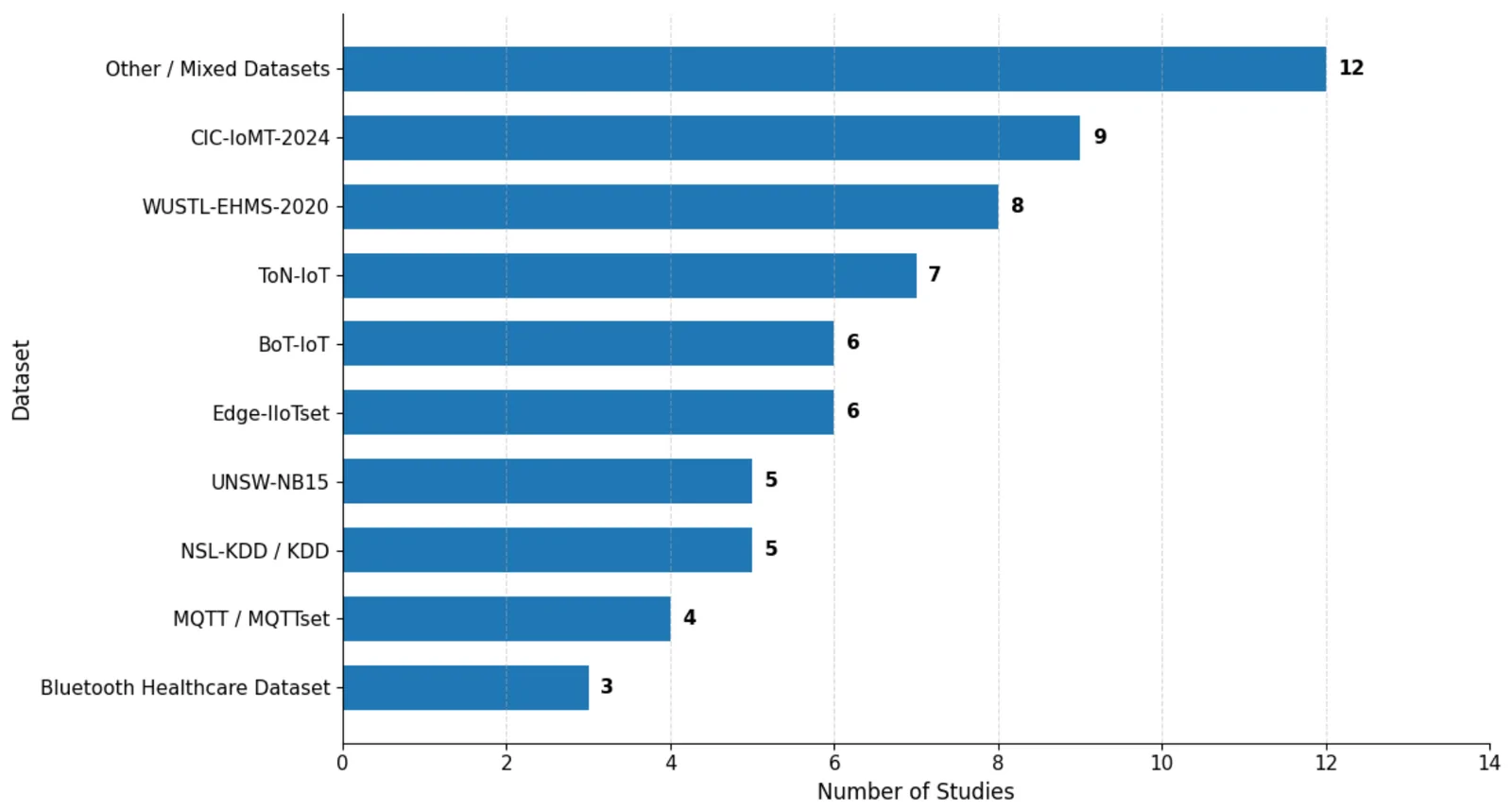
Distribution of benchmark datasets used across the reviewed AI-powered IoMT intrusion detection studies.

Figure 2 illustrates the distribution of benchmark datasets used across the reviewed studies. The concentration of evaluations on datasets such as WUSTL-EHMS-2020, CIC-IoMT-2024, ToN-IoT, BoT-IoT, Edge-IIoTset, and CIC-IDS2017 highlights both the value of shared benchmarks and the risk of dataset dependency. This reinforces the need for healthcare-specific datasets, standardized evaluation protocols, and rigorous cross-dataset validation.

Collectively, the thematic classification, detailed study extraction, and dataset distribution analysis establish the evidence base for the comparative synthesis presented in Section 3.

### 2.4. Data Extraction and Synthesis

A standardized data extraction framework was used to ensure consistency, transparency, and comparability across the 24 primary studies retained after eligibility screening and quality assessment. Using a predefined comparative synthesis matrix, data were extracted on benchmark datasets, AI and IDS methods, intrusion detection objectives, architectural characteristics, explainability mechanisms, privacy-preserving approaches, evaluation metrics, principal findings, limitations, and future research directions.

Only the 24 primary studies were included in the formal methodological comparison and evidence synthesis. The remaining 115 supporting studies were used to strengthen the conceptual background, methodological justification, Healthcare 5.0 and Remote Patient Monitoring (RPM) context, and interpretation of emerging research trends, but were not included in the formal comparative synthesis or quality assessment.

The extracted evidence was synthesized across six analytical dimensions: detection performance, explainability, privacy preservation, computational efficiency, benchmarking practices, and cross-dataset generalization. These dimensions enabled comparison across heterogeneous study designs while supporting the identification of recurring strengths, limitations, trends, and research gaps.

Consistent with the structured narrative and qualitative character of this review, a quantitative meta-analysis was not conducted because the included studies differed substantially in datasets, attack categories, AI architectures, evaluation metrics, experimental settings, and validation protocols. Instead, a structured qualitative comparative synthesis was adopted to preserve methodological context while enabling critical comparison across studies. This synthesis framework provides the methodological foundation for the thematic analysis in Section 3, the critical discussion in Section 4, and the future research directions in Section 5.

Although only 24 studies were retained as the primary evidence base, this number is appropriate for the objectives of the present review and should be interpreted within the context of the study selection process. The reduction from 5127 initially identified records to 24 included studies primarily reflects scope refinement and relevance-based screening rather than widespread exclusion due to methodological quality. The majority of excluded records addressed general Internet of Things security, clinical artificial intelligence applications without a cybersecurity focus, or healthcare technologies beyond the scope of AI-powered IoMT intrusion detection, and therefore were not suitable for the comparative synthesis. Among the 34 studies that reached full-text eligibility—i.e., studies already confirmed to be on-topic—24 (71%) met the quality threshold. The six analytical dimensions adopted in this review were used as a common analytical framework to evaluate the same carefully selected body of evidence rather than as independent evidence syntheses. Furthermore, the additional 115 supporting studies were incorporated to provide conceptual, methodological, and technological context, while the formal comparative synthesis remained intentionally restricted to the most relevant and methodologically robust primary studies. Collectively, the included studies encompass diverse AI techniques, benchmark datasets, explainability methods, privacy-preserving approaches, deployment scenarios, and Healthcare 5.0 applications, providing sufficient thematic breadth to identify consistent research trends, recurring limitations, and common deployment challenges across the literature. Accordingly, the objective of this review was to achieve analytical depth and methodological rigor through a focused qualitative synthesis rather than maximize the number of included studies.

## 3. Literature Review and Thematic Analysis

### 3.1. Evolution of AI-Powered Intrusion Detection Systems for IoMT

The evolution of Intrusion Detection Systems (IDSs) for the Internet of Medical Things (IoMT) reflects a fundamental transformation from static attack detection toward intelligent, trustworthy, and deployment-oriented cybersecurity. This transformation has been driven by the rapid expansion of interconnected healthcare ecosystems, where wearable sensors, implantable medical devices, Remote Patient Monitoring (RPM) platforms, cloud–edge infrastructures, and Healthcare 5.0 services have simultaneously enhanced clinical capabilities and enlarged the healthcare cyberattack surface [4,6,10,11,12,53]. Consequently, IDSs have evolved from auxiliary security mechanisms into essential components of resilient healthcare infrastructures responsible for safeguarding data confidentiality, system integrity, service availability, and patient safety across increasingly distributed clinical environments [17,22,23,33,48,54,55,56].

**Figure 3:**
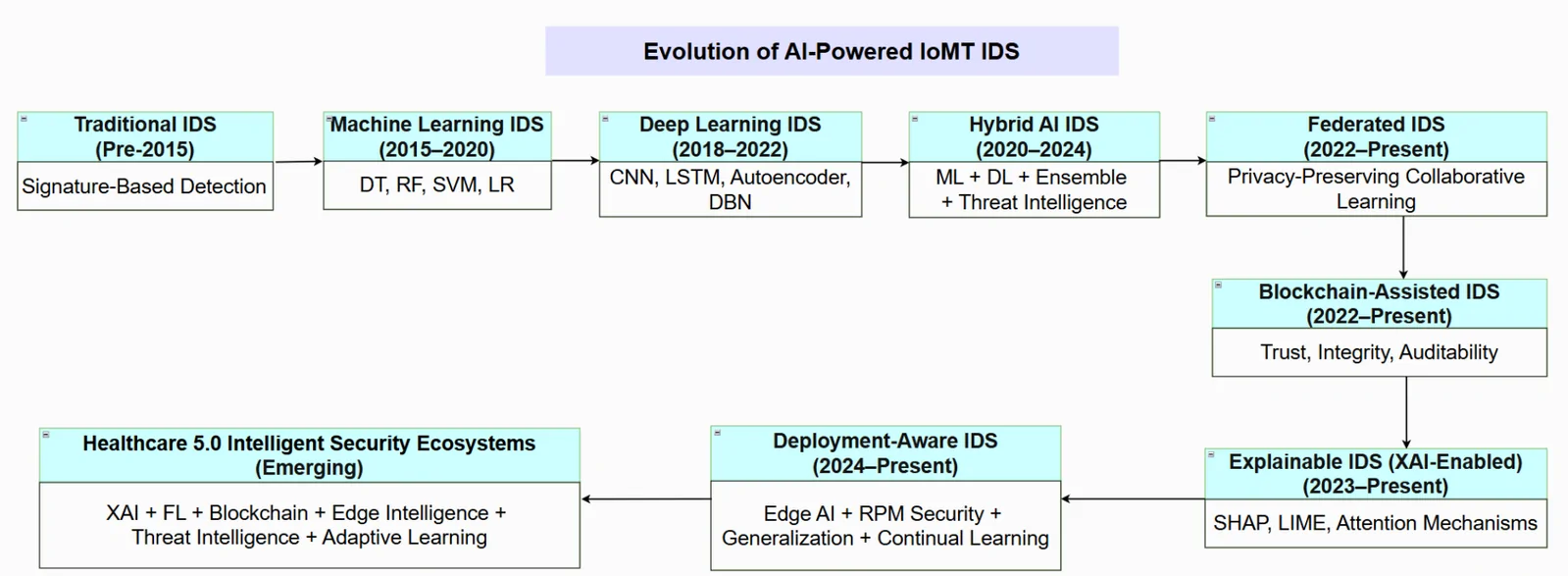
Conceptual evolution of AI-powered intrusion detection systems for the Internet of Medical Things (IoMT). The progression extends beyond algorithmic advancement, illustrating the transformation from signature-based detection toward intelligent, trustworthy, privacy-preserving, explainable, and deployment-ready cybersecurity architectures. The figure highlights how research priorities have evolved from maximizing detection accuracy to balancing behavioural intelligence, operational scalability, computational efficiency, privacy preservation, trust management, and real-world deployment requirements within Healthcare 5.0 ecosystems.

Rather than representing a simple progression of increasingly sophisticated algorithms, the evolution of IoMT intrusion detection has been driven by successive attempts to overcome the limitations of preceding security paradigms. As illustrated in Figure 3, this evolution can be understood through three major transitions. The first relied on signature- and rule-based IDSs, which effectively detected known attacks using predefined signatures and handcrafted rules but were inherently unable to adapt to zero-day attacks, polymorphic malware, insider threats, or rapidly evolving attack behaviours. These shortcomings became increasingly pronounced in heterogeneous IoMT environments, where constrained devices, diverse communication protocols, continuous medical data streams, and dynamic network conditions reduced scalability while increasing false-positive rates [11,17,21,48,57,58,59].

The inability of static detection mechanisms to address emerging cyber threats accelerated the adoption of Artificial Intelligence (AI), marking the second major stage in IoMT IDS evolution [20,36]. Classical machine learning (ML) algorithms—including Decision Trees (DT), Random Forests (RF), Support Vector Machines (SVMs), Logistic Regression (LR), and Extreme Gradient Boosting (XGBoost)—enabled IDSs to learn behavioural patterns directly from heterogeneous network traffic, substantially improving the detection of previously unseen attacks without relying on manually defined rules [24,25,37,38,39,47,60,61,62]. However, evidence across the reviewed literature consistently indicates that the dependence of conventional ML on handcrafted feature engineering and dataset-specific representations limited cross-dataset generalization and robustness in heterogeneous healthcare environments, motivating the transition toward deep learning (DL) approaches [40,41,59,63].

The third stage of this evolution was characterized by the widespread adoption of DL architectures capable of automatically learning hierarchical spatial, temporal, and behavioural representations from complex IoMT traffic [60,64,65]. Architectures such as Convolutional Neural Networks (CNNs), Long Short-Term Memory (LSTM) networks, Autoencoders, Deep Belief Networks (DBNs), Transformers, and more recently hybrid and ensemble models consistently demonstrated superior detection performance across benchmark datasets by reducing dependence on manual feature engineering and capturing increasingly sophisticated attack patterns [29,30,61,65,66,67,68,69,70]. Nevertheless, the reviewed evidence also demonstrates that improved predictive performance has not fully resolved challenges related to computational complexity, model interpretability, cross-dataset robustness, and deployment under realistic healthcare conditions. These limitations have stimulated growing interest in hybrid AI architectures that combine complementary learning paradigms to improve operational reliability while supporting more practical deployment scenarios [71,72,73,74,75,76,77,78].

Importantly, the contemporary evolution of IoMT intrusion detection extends beyond algorithmic innovation. The literature demonstrates a clear shift from maximizing detection accuracy toward developing trustworthy, scalable, and clinically deployable security frameworks that simultaneously address explainability, privacy preservation, interoperability, computational efficiency, and operational resilience [15,16,77]. This transition has accelerated the integration of complementary technologies—including Explainable Artificial Intelligence (XAI), Federated Learning (FL), blockchain-assisted trust management, edge intelligence, and collaborative learning—which collectively seek to balance predictive capability with transparency, secure knowledge sharing, regulatory compliance, and deployment readiness in distributed healthcare ecosystems [1,23,31,32,34,35,79,80,81,82,83,84,85,86,87]. Rather than representing independent technological advances, these developments collectively reflect a broader transition from algorithm-centred intrusion detection toward deployment-oriented cybersecurity capable of supporting the operational requirements of Healthcare 5.0.

Overall, the reviewed literature indicates that the evolution of IoMT IDSs has been shaped by three interconnected paradigm shifts: from static signature matching to adaptive AI-driven behavioural intelligence, from centralized detection to collaborative and privacy-preserving security architectures, and from benchmark-oriented evaluation to deployment-oriented assessment emphasizing robustness, explainability, scalability, computational efficiency, and cross-dataset generalization [24,36,40,41,56,62,88,89,90]. These shifts demonstrate that future progress will depend less on developing increasingly complex prediction models than on integrating trustworthy AI, collaborative intelligence, and deployment-aware system design into unified cybersecurity frameworks capable of supporting secure, resilient, and clinically applicable IoMT ecosystems.

Figure 3 summarizes this evolutionary trajectory and establishes the conceptual foundation for the thematic analysis presented in the following subsections, where the major AI paradigms, enabling technologies, and remaining deployment challenges are examined in greater depth.

### 3.2. Detection Performance: From Machine Learning to Hybrid AI

Building on the evolutionary trends discussed in Section 3.1, advances in detection performance have been the principal catalyst driving successive generations of AI-powered intrusion detection systems (IDSs) for the Internet of Medical Things (IoMT). As healthcare environments have become increasingly heterogeneous, distributed, and data-intensive, conventional signature-based mechanisms proved inadequate for detecting sophisticated and evolving cyberattacks, accelerating the transition toward Artificial Intelligence (AI)-driven IDSs [12]. However, the reviewed literature demonstrates that the evolution of detection performance has not been driven solely by the pursuit of higher predictive accuracy. Instead, each generation of AI techniques emerged to overcome limitations of its predecessor while introducing new trade-offs that shaped subsequent research directions [20,22,23,29,36,55,62,91].

The first generation of AI-powered IoMT IDSs was dominated by traditional machine learning (ML), where supervised algorithms learned behavioural patterns directly from network traffic rather than relying on predefined attack signatures. Algorithms such as Decision Trees (DT), Random Forests (RF), Support Vector Machines (SVMs), Logistic Regression (LR), and Extreme Gradient Boosting (XGBoost) consistently improved the detection of previously unseen attacks while maintaining relatively low computational overhead, making them attractive for resource-constrained healthcare environments [25,47,48,60,61]. Among these approaches, tree-based ensemble models, particularly Random Forest and XGBoost, achieved a favourable balance between detection performance, computational efficiency, robustness, and interpretability [26,27,73]. Nevertheless, their dependence on handcrafted feature engineering and dataset-specific representations constrained their ability to generalize across heterogeneous healthcare environments, motivating the transition toward deep learning architectures [24,40,41,59].

Deep learning (DL) addressed many of these limitations by replacing manual feature engineering with automated hierarchical representation learning, enabling IDSs to capture complex spatial, temporal, and behavioural characteristics of healthcare network traffic [64,65,92]. Architectures including Convolutional Neural Networks (CNNs), Long Short-Term Memory (LSTM) networks, Autoencoders, Deep Belief Networks (DBNs), and Transformer-based models consistently demonstrated superior detection capability across widely used benchmark datasets by modelling increasingly sophisticated attack patterns [29,30,66,67,68,69,93]. Numerous studies reported detection accuracies exceeding 95% on benchmark datasets such as CIC-IoMT-2024 and WUSTL-EHMS-2020 using CNN-LSTM, Transformer-enhanced, and deep stacking architectures [28,61,68,70,75,76,91,94,95]. However, the reviewed evidence indicates that these improvements were frequently accompanied by increased computational complexity, reduced model transparency, and greater implementation overhead, highlighting that higher predictive performance alone does not necessarily translate into more practical intrusion detection systems [29,40,41,96,97].

To balance the complementary strengths and weaknesses of individual learning paradigms, recent research has increasingly adopted hybrid and ensemble AI architectures that integrate multiple modelling techniques within a unified detection framework. Rather than seeking incremental improvements from individual algorithms, these approaches combine complementary capabilities such as automated feature extraction, temporal dependency modelling, ensemble decision making, and adaptive learning to improve robustness, predictive stability, and attack coverage across diverse cyber-threat scenarios. Representative architectures—including CNN-LSTM, Transformer-enhanced, Autoencoder-based, graph-enhanced, stacking, and hybrid ensemble frameworks—consistently outperform individual ML or DL models by exploiting synergistic learning strategies instead of relying on a single classifier [70,71,73,74,75,76,77,78,95,97,98,99]. Collectively, these findings indicate that recent advances in IoMT intrusion detection are increasingly driven by architectural integration rather than the development of entirely new learning algorithms.

**Figure 4:**
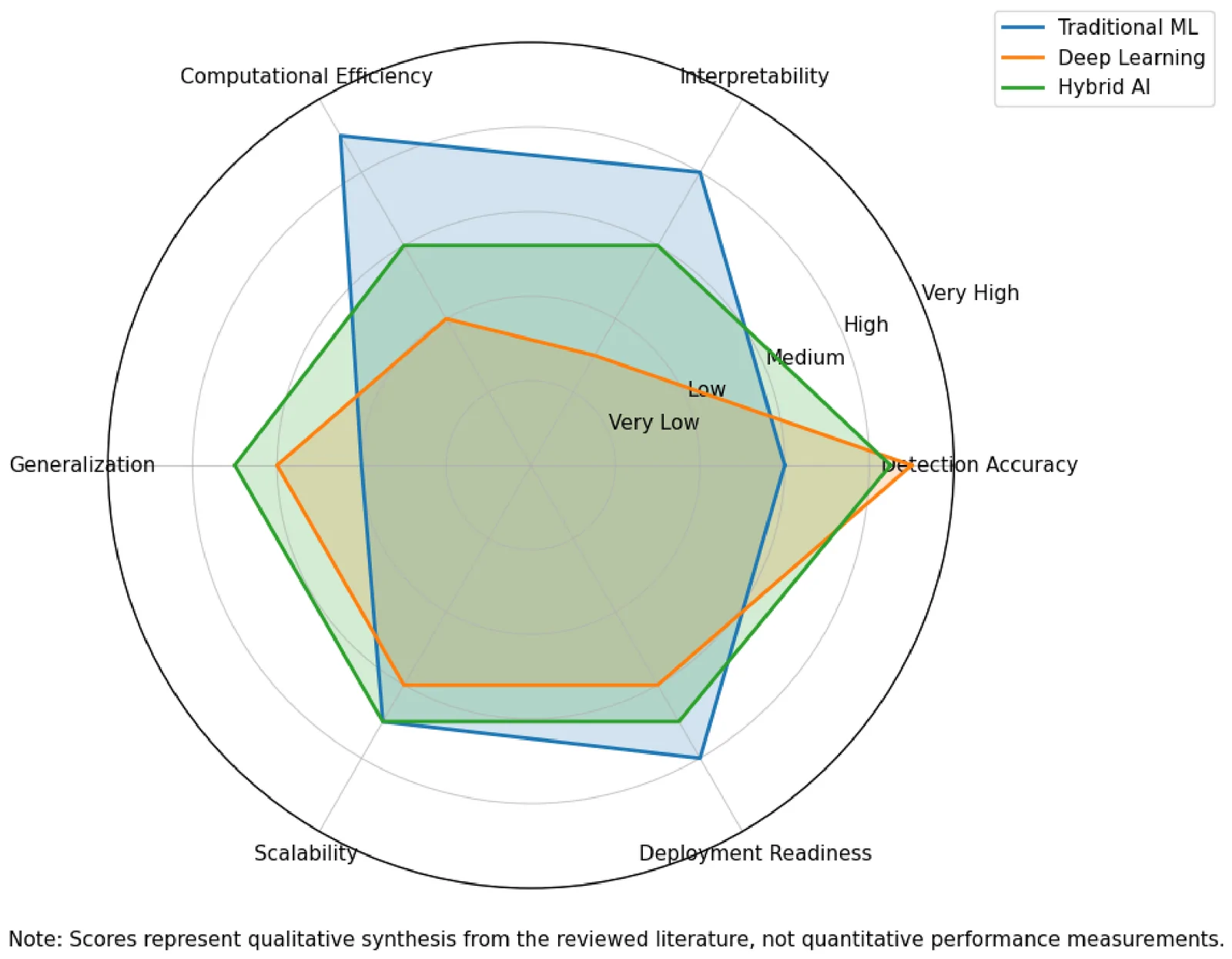
Conceptual comparison of major AI paradigms across deployment-oriented dimensions for IoMT intrusion detection systems. The figure presents an illustrative qualitative synthesis of the reviewed literature and summarizes the relative trade-offs among detection capability, interpretability, computational efficiency, scalability, generalization, and deployment readiness. It is intended as a conceptual visual summary rather than a quantitative evaluation or formal scoring system.

Table 8 and Figure 4 synthesize the principal characteristics of the major AI paradigms identified across the reviewed literature. Figure 4 is intended as an illustrative qualitative synthesis of the recurring patterns identified across the reviewed studies and should be interpreted as a conceptual visual summary rather than a quantitative evaluation or formal scoring system. Rather than identifying a universally superior approach, the evidence demonstrates that each paradigm emerged to address shortcomings of the previous generation while introducing new technical and operational trade-offs. Traditional ML offers computational efficiency and relatively high interpretability but remains dependent on handcrafted features; DL substantially improves feature learning and detection capability at the cost of increased computational demand and reduced transparency; hybrid and ensemble architectures seek to balance these competing objectives through complementary learning strategies, whereas adaptive learning extends this progression by addressing concept drift and evolving attack behaviours despite limited large-scale validation [89]. Consequently, selecting an AI paradigm should be regarded as a deployment-driven engineering decision that balances detection capability with computational resources, latency requirements, scalability, and operational constraints rather than maximizing benchmark accuracy alone.

Overall, the reviewed evidence demonstrates that improvements in detection performance have been achieved through progressively richer feature representations and increasingly integrated learning architectures rather than through isolated algorithmic innovation. Nevertheless, no individual paradigm simultaneously satisfies the competing requirements of predictive accuracy, computational efficiency, interpretability, scalability, and operational robustness. This observation suggests that future advances in IoMT intrusion detection will depend less on developing more complex detection algorithms than on designing balanced AI architectures capable of supporting trustworthy and practical healthcare cybersecurity. These trade-offs naturally motivate the following discussion on Explainable Artificial Intelligence (XAI), where model transparency and stakeholder trust become essential for translating high-performing AI models into clinically acceptable intrusion detection systems.

### 3.3. Explainable and Trustworthy AI for IoMT Intrusion Detection

Although Artificial Intelligence (AI) has substantially improved intrusion detection performance in the Internet of Medical Things (IoMT), predictive accuracy alone is insufficient for safe and reliable deployment in healthcare environments. Contemporary machine learning, deep learning, and hybrid AI models frequently report detection accuracies exceeding 95% on benchmark datasets; however, these metrics provide limited assurance that clinicians, cybersecurity analysts, and healthcare administrators can understand, validate, and confidently act upon AI-generated security decisions [15,19,22,23,24,29]. Consequently, the literature demonstrates a clear shift from maximizing detection capability toward developing explainable and trustworthy AI systems that combine accurate threat detection with transparency, accountability, human oversight, and regulatory compliance.

This transition is particularly important in healthcare because intrusion detection decisions may directly influence clinical workflows, connected medical devices, Remote Patient Monitoring (RPM) services, and ultimately patient safety. False-positive alerts can disrupt healthcare operations and increase alert fatigue, whereas false negatives may expose sensitive medical information or compromise life-critical systems. Accordingly, explainability has evolved from a desirable model characteristic into a prerequisite for trustworthy cybersecurity by enabling stakeholders to understand why alerts are generated and whether they should be trusted in operational environments [2,5,13,14,16,48,55,100,101,102,103].

**Figure 5:**
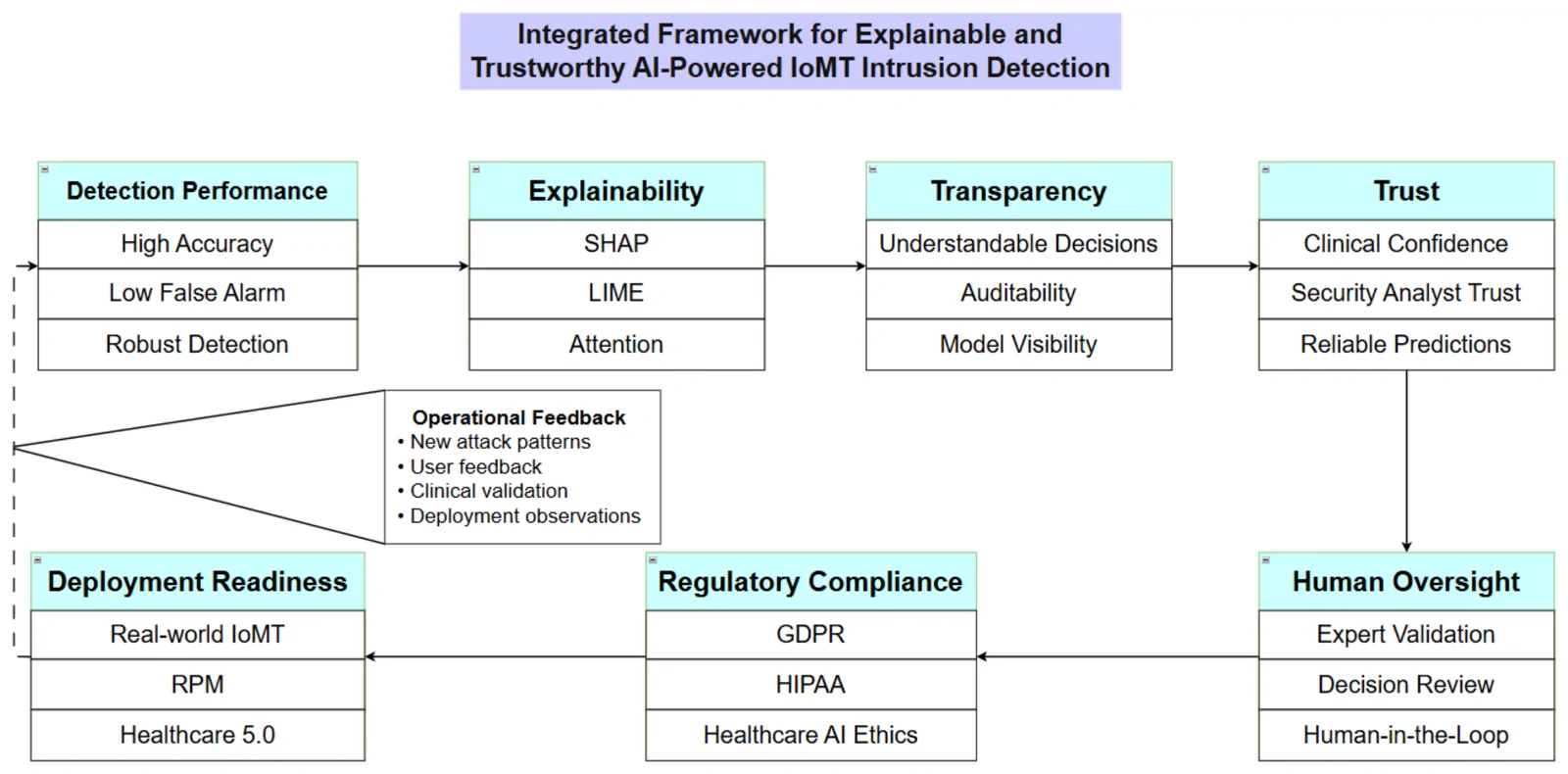
Integrated framework for trustworthy AI-powered IoMT intrusion detection. Detection performance forms the technical foundation, while explainability enables transparency, stakeholder trust, human oversight, regulatory compliance, and ultimately deployment-ready Healthcare 5.0 cybersecurity.

Figure 5 illustrates this conceptual evolution. Rather than representing independent objectives, detection performance, explainability, stakeholder trust, accountability, governance, and human oversight form complementary components of a unified deployment framework. Within this framework, predictive performance provides the technical foundation, whereas explainability enables transparent decision-making that strengthens stakeholder confidence and supports the broader objectives of trustworthy AI. The dotted arrow represents an operational feedback loop from real-world deployment back to detection performance. This feedback includes new attack patterns, user feedback, clinical validation, and deployment observations.

Explainable Artificial Intelligence (XAI) provides the practical mechanisms through which AI-generated security decisions become interpretable. Rather than functioning solely as post-processing tools, XAI techniques enable analysts to identify the factors influencing model predictions, distinguish genuine cyber threats from spurious detections, and improve confidence in AI-assisted decision making [77,104]. Across the reviewed literature, five principal explainability paradigms dominate IoMT intrusion detection: SHapley Additive exPlanations (SHAP), Local Interpretable Model-Agnostic Explanations (LIME), attention mechanisms, intrinsically interpretable models, and explainable ensemble approaches [26,77,105,106,107,108]. Although these methods differ in implementation, they illustrate a common progression from explaining model outputs after prediction toward embedding transparency directly within model design. SHAP and LIME provide global and local post-hoc explanations with different trade-offs between explanation fidelity and computational cost, whereas attention mechanisms and intrinsically interpretable models integrate transparency into the learning process itself. Explainable ensemble approaches further seek to balance predictive performance with interpretable decision making [93,99]. Consequently, the reviewed evidence indicates that selecting an XAI technique is fundamentally a deployment-oriented design decision that must consider computational efficiency, scalability, regulatory requirements, and analyst usability alongside predictive performance.

Table 9 summarizes the principal explainability techniques and their complementary strengths and limitations. Collectively, these studies demonstrate that explainability is no longer an optional enhancement but an essential requirement for AI-powered IoMT intrusion detection.

While explainability substantially improves transparency, the reviewed literature consistently demonstrates that it alone cannot guarantee safe clinical deployment. Post-hoc explanations may clarify why individual predictions are made, but they provide limited assurance regarding model reliability, robustness, fairness, privacy preservation, governance, or resilience against evolving cyber threats [22,23,24,77]. As a result, contemporary IoMT cybersecurity research increasingly positions explainability as the foundation of a broader trustworthy AI framework rather than the final objective of intelligent intrusion detection.

Trustworthy AI extends explainability by integrating complementary technical, operational, and human-centred properties—including reliability, robustness, fairness, privacy preservation, accountability, governance, and meaningful human oversight—into a unified deployment paradigm [2,34,69,86,87,100,101,109]. Rather than representing independent evaluation criteria, these dimensions collectively determine whether AI-powered IDSs can maintain dependable performance under evolving attack scenarios while providing transparent and clinically defensible security recommendations.

Despite substantial progress, important challenges remain. Most existing studies continue to emphasize benchmark detection performance while providing comparatively limited evaluation of long-term reliability, adversarial robustness, fairness across heterogeneous healthcare environments, AI governance, and human-centred usability. Likewise, standardized methodologies for evaluating trustworthy AI remain underdeveloped, making it difficult to compare competing approaches or assess their readiness for operational healthcare deployment [1,24,100,101,109,110,111,112,113,114].

Table 10 synthesizes the principal dimensions of trustworthy AI identified across the reviewed literature.

The reviewed literature demonstrates a clear conceptual progression from explainable AI toward comprehensive trustworthy AI frameworks. Whereas early research primarily focused on improving model interpretability, contemporary studies increasingly recognize that transparent decision making represents only one component of clinically deployable intrusion detection. Effective IoMT cybersecurity requires integrating explainability with reliability, robustness, privacy preservation, fairness, governance, accountability, and meaningful human oversight to establish confidence in AI-assisted security decisions.

Collectively, these findings indicate that explainability should be regarded as the enabling mechanism through which trustworthy AI is achieved rather than as an independent research objective. This evolution naturally extends the focus of IoMT intrusion detection from transparent decision support toward collaborative and privacy-preserving intelligence, motivating the following discussion on Federated Learning (FL), blockchain-assisted security, and distributed AI for healthcare cybersecurity.

### 3.4. Collaborative and Privacy-Preserving AI for IoMT Intrusion Detection

The rapid adoption of AI-powered intrusion detection systems (IDSs) in the Internet of Medical Things (IoMT) has fundamentally changed the requirements for healthcare cybersecurity. Beyond achieving high detection performance, contemporary IDSs must enable secure collaboration among hospitals, wearable devices, Remote Patient Monitoring (RPM) platforms, cloud services, and edge infrastructures while preserving patient privacy, institutional autonomy, and regulatory compliance. Because centralized learning requires sensitive healthcare data to be aggregated into shared repositories, recent research has increasingly shifted toward collaborative AI architectures that support distributed model development without exposing raw patient information [21,31,32,34,35,79,81,83,84]. This transition represents a broader evolution from centralized intelligence toward privacy-preserving and trust-enabled cybersecurity capable of supporting Healthcare 5.0 ecosystems.

**Figure 6:**
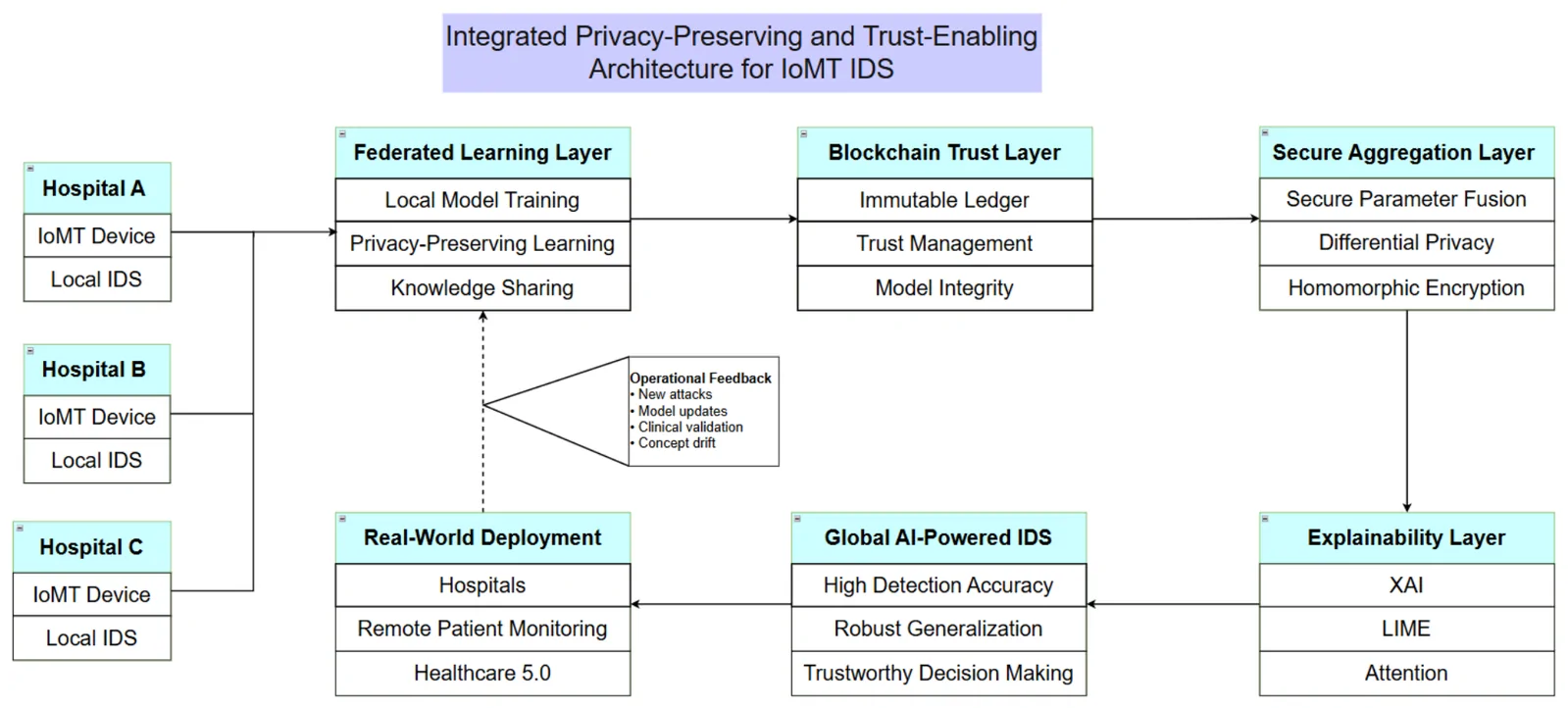
Integrated privacy-preserving and trust-enabling architecture for AI-powered IoMT intrusion detection systems. Federated Learning enables collaborative model training without sharing raw patient data, blockchain establishes decentralized trust and secure aggregation, while Explainable AI provides transparency and human oversight. Together, these complementary technologies support deployment-ready Healthcare 5.0 cybersecurity.

Figure 6 illustrates the integrated architecture emerging from the reviewed literature. Rather than functioning as independent technologies, Federated Learning (FL), blockchain, and Explainable Artificial Intelligence (XAI) operate as complementary architectural layers. Federated Learning enables collaborative model training while retaining sensitive healthcare data within local institutions, blockchain establishes decentralized trust and secure collaboration, and XAI provides transparent decision support for human stakeholders. Together, these technologies balance privacy preservation, accountability, transparency, and operational resilience within distributed healthcare environments. The dotted arrow represents an operational feedback loop from real-world deployment to the federated learning layer. The feedback includes new attacks, drift indicators, user feedback, and compliance-related information.

Federated Learning has emerged as the dominant collaborative learning paradigm because it enables multiple healthcare organizations to train shared intrusion detection models while keeping patient data within local institutions. This decentralized learning strategy improves regulatory compliance, preserves data sovereignty, and exposes AI models to more diverse operational environments than isolated institutional datasets, thereby increasing their potential robustness and generalizability [31,32,79,83,84,115]. Nevertheless, the reviewed literature consistently identifies several obstacles to large-scale deployment. Highly heterogeneous (Non-IID) healthcare data reduce aggregation effectiveness, repeated communication introduces latency and computational overhead, and collaborative training remains vulnerable to poisoning attacks, membership inference, model inversion, and malicious participants [32,110,111,112,116,117,118,119,120,121,122,123]. Consequently, current research increasingly focuses on personalized federated learning, adaptive aggregation, communication-efficient optimization, secure aggregation, adversarial resilience, and AI-enhanced cyber threat intelligence to improve both scalability and trustworthiness [88,124,125].

While Federated Learning protects data privacy, it does not by itself establish trustworthy collaboration among autonomous healthcare organizations. Blockchain therefore complements FL by providing immutable audit trails, decentralized identity management, secure provenance tracking, participant authentication, and transparent verification of collaborative model updates rather than functioning as an independent intrusion detection mechanism [34,35,44,45,80,85]. Together, FL and blockchain address complementary challenges: FL preserves data confidentiality through decentralized learning, whereas blockchain strengthens accountability, secure coordination, and resilience against collaborative attacks [34,44,79,126]. Despite these advantages, practical deployment remains constrained by consensus latency, communication overhead, interoperability across heterogeneous IoMT infrastructures, and resource limitations, motivating lightweight consensus mechanisms and hybrid on-chain/off-chain architectures that store only cryptographic verification data on-chain while retaining sensitive healthcare information locally [53,56,81,85,127,128,129].

Table 11 summarizes the complementary roles of Federated Learning, blockchain, secure aggregation, and Explainable AI within collaborative IoMT intrusion detection. Rather than representing competing technologies, these approaches collectively demonstrate that deployment-ready Healthcare 5.0 cybersecurity depends on integrating privacy preservation, decentralized trust, transparent decision support, and efficient collaborative learning within a unified architectural framework.

The reviewed literature demonstrates a clear transition from isolated AI techniques toward integrated collaborative cybersecurity architectures. Rather than relying on Federated Learning, blockchain, or Explainable AI independently, contemporary IoMT IDSs increasingly combine these complementary technologies to address privacy preservation, decentralized trust, transparency, and secure collaboration simultaneously. Nevertheless, challenges associated with Non-IID data, adversarial robustness, communication efficiency, interoperability, and standardized evaluation remain unresolved. These findings indicate that future advances will depend less on developing individual collaborative technologies than on designing unified architectures capable of balancing privacy, trust, scalability, and operational efficiency, providing the foundation for the following discussion on deployment readiness, benchmarking practices, computational efficiency, and cross-dataset generalization.

### 3.5. Deployment Readiness and Real-World Implementation Challenges

Although recent advances in machine learning, deep learning, explainable AI, and collaborative intelligence have substantially improved IoMT intrusion detection, the reviewed literature consistently demonstrates that operational deployment continues to lag behind algorithmic progress. This gap reflects not a lack of detection capability but the complexity of deploying AI-powered IDSs across heterogeneous healthcare ecosystems comprising wearable devices, Remote Patient Monitoring (RPM) platforms, cloud–edge infrastructures, diverse medical devices, and regulatory environments [1,2,13,14,16,22,23,24,29,52,102,103,130]. Collectively, the reviewed evidence indicates that deployment readiness has become the defining criterion for translating laboratory success into clinically applicable cybersecurity.

A consistent finding across the literature is the growing disconnect between benchmark performance and operational effectiveness. Although contemporary AI-powered IDSs frequently report detection accuracies exceeding 95% on benchmark datasets such as CIC-IoMT-2024, WUSTL-EHMS-2020, ToN-IoT, MQTT-based healthcare datasets, and Edge-IIoTset [26,27,28,73,74,75,76,94,95], reviews consistently caution that these results often overestimate real-world performance because evaluation protocols remain dominated by single datasets, static train–test partitions, balanced attack distributions, and synthetic traffic [22,23,24,40,41,59]. Consequently, benchmark accuracy should be interpreted as evidence of algorithmic capability rather than operational readiness.

Rather than representing isolated limitations, the reviewed studies identify four interrelated barriers that collectively constrain real-world deployment: limited cross-dataset generalization, computational constraints, dynamic operating environments, and insufficient integration with clinical workflows.

Cross-dataset generalization remains the most widely reported challenge. Models trained on individual benchmark datasets frequently experience substantial performance degradation when evaluated across different healthcare environments, indicating that many AI models learn dataset-specific characteristics rather than generalized representations of malicious behaviour [22,24,40,41,59]. These limitations arise from differences in medical devices, communication protocols, traffic characteristics, feature engineering pipelines, and attack distributions across healthcare infrastructures, highlighting the need for external validation, domain adaptation, transfer learning, and multi-centre benchmarking rather than continued optimization for single datasets [1,2,5,7,8,13,16,131,132].

Computational efficiency represents a second major barrier. While deep learning and hybrid architectures consistently improve detection capability, they also introduce greater processing, memory, latency, and energy requirements that remain difficult to satisfy on wearable devices, implantable sensors, edge gateways, and battery-powered RPM platforms [21,29,75,81,94,95,96]. Consequently, lightweight deep learning, TinyML, model compression, pruning, knowledge distillation, and edge intelligence have emerged as promising directions for balancing detection capability with resource efficiency [1,29,133,134].

The reviewed literature further indicates that most existing IDSs remain optimized for static offline learning despite operating within continuously evolving healthcare environments. Concept drift, software updates, changing communication patterns, and emerging cyber threats gradually reduce detection performance, motivating increasing interest in continual learning, incremental learning, adaptive intelligence, and transfer learning to support long-term operational reliability [52,62,135,136,137].

Finally, successful deployment depends on effective integration with clinical workflows and deployment-oriented evaluation. Current benchmarking practices continue to emphasize accuracy-based metrics while providing comparatively limited evidence regarding latency, energy consumption, explainability, interoperability, adversarial robustness, usability, regulatory compliance, and long-term operational reliability [22,23,24,40,52]. Consequently, AI-powered IDSs should be evaluated as clinical decision-support systems rather than standalone classification models, emphasizing transparent recommendations that complement clinicians and cybersecurity analysts instead of replacing them [1,2,5,15,23,48,138].

Table 12 synthesizes the principal deployment barriers and corresponding research priorities identified across the reviewed literature, while Table 13 summarizes the practical criteria required to evaluate deployment readiness under realistic Healthcare 5.0 conditions.

The reviewed evidence demonstrates that deployment readiness has become the principal challenge for AI-powered IoMT intrusion detection. Rather than requiring fundamentally new detection algorithms, future progress depends on demonstrating that existing AI models can generalize across heterogeneous healthcare environments, operate efficiently on resource-constrained platforms, adapt to evolving cyber threats, integrate seamlessly with clinical workflows, and satisfy multidimensional deployment requirements extending beyond predictive accuracy. Collectively, these findings establish deployment-oriented validation as the critical bridge between laboratory performance and trustworthy real-world healthcare cybersecurity, providing the foundation for the future research directions presented in the following section.

### 3.6. Critical Research Gaps in AI-Powered IoMT Intrusion Detection

The preceding synthesis demonstrates that AI-powered intrusion detection systems (IDSs) for the Internet of Medical Things (IoMT) have advanced substantially in detection capability, explainability, privacy preservation, collaborative learning, and deployment-oriented design. However, the reviewed literature indicates that these advances remain fragmented and have not yet converged into mature cybersecurity solutions suitable for routine healthcare deployment. Rather than reflecting isolated technical limitations, the remaining challenges are interconnected and require system-level solutions that integrate robust AI, trustworthy decision-making, privacy-preserving collaboration, and operational validation within modern healthcare ecosystems [1,2,18,19,22,23,24,52,86,87].

Across the reviewed studies, five major research gaps consistently emerge. First, many IDSs remain dependent on benchmark-specific learning, resulting in limited generalization across unseen datasets, institutions, devices, and communication environments [24,40,41,59,135,137]. Second, computationally intensive deep learning and hybrid architectures remain difficult to deploy on wearable sensors, implantable devices, edge gateways, and Remote Patient Monitoring platforms, where memory, latency, and energy constraints are critical [1,29,81,96,130,131,132,133,134]. Third, collaborative intelligence remains constrained by Non-IID data, communication overhead, poisoning attacks, trust management, and scalability limitations despite growing interest in Federated Learning, blockchain, secure aggregation, and privacy-preserving optimization [31,32,34,44,46,88,115,124,125,126].

Fourth, trustworthy AI remains insufficiently integrated into IoMT IDS design. Although recent studies increasingly adopt explainability methods such as SHAP, LIME, attention mechanisms, and interpretable ensemble models, many systems still provide limited evidence of robustness, fairness, governance, regulatory compliance, and meaningful human oversight [2,26,27,105,106,113,114]. Fifth, current evaluation methodologies remain overly focused on accuracy, precision, recall, F1-score, and ROC–AUC, while providing limited assessment of latency, energy consumption, interoperability, scalability, adversarial resilience, clinical usability, and long-term operational reliability under realistic healthcare conditions [22,23,52].

Collectively, these gaps demonstrate that the central limitation of current IoMT IDS research is not the absence of high-performing AI models, but the lack of integrated, validated, and deployment-ready cybersecurity systems. Improvements in model generalization have limited practical value without resource-efficient implementation; collaborative intelligence remains incomplete without trustworthy governance; and benchmark performance remains insufficient without external validation under realistic healthcare conditions. Therefore, the reviewed evidence indicates that future progress depends on moving from isolated algorithmic innovation toward integrated system design that jointly addresses generalization, efficiency, privacy preservation, trustworthiness, interoperability, and operational evaluation.

The evidence synthesized in this section shows that AI-powered IoMT intrusion detection is approaching a transition point. The field has produced increasingly accurate, explainable, privacy-preserving, and collaborative models, yet these capabilities remain insufficiently connected to real-world healthcare requirements. The next stage of research must therefore focus on closing the gap between laboratory performance and operational deployment by validating IDSs across heterogeneous healthcare environments, resource-constrained devices, evolving threat conditions, and clinically meaningful workflows. These gaps provide the foundation for the future research directions presented in Section 5.

## 4. Discussion

### 4.1. From Algorithm-Centred to System-Centred IoMT Intrusion Detection

The evidence synthesized throughout this review demonstrates that the evolution of Artificial Intelligence (AI)-powered intrusion detection for the Internet of Medical Things (IoMT) extends well beyond improvements in predictive accuracy. Earlier research primarily focused on developing increasingly sophisticated machine learning and deep learning models capable of detecting complex cyberattacks. However, contemporary studies consistently indicate that successful healthcare cybersecurity depends equally on explainability, privacy preservation, trustworthiness, scalability, computational efficiency, interoperability, and operational deployment. Collectively, these findings reveal a fundamental transition from algorithm-centred IDS development toward integrated cybersecurity systems capable of supporting Remote Patient Monitoring (RPM), digital healthcare services, and Healthcare 5.0 ecosystems [2,13,15,16,22,23,24,29,52,103].

**Figure 7:**
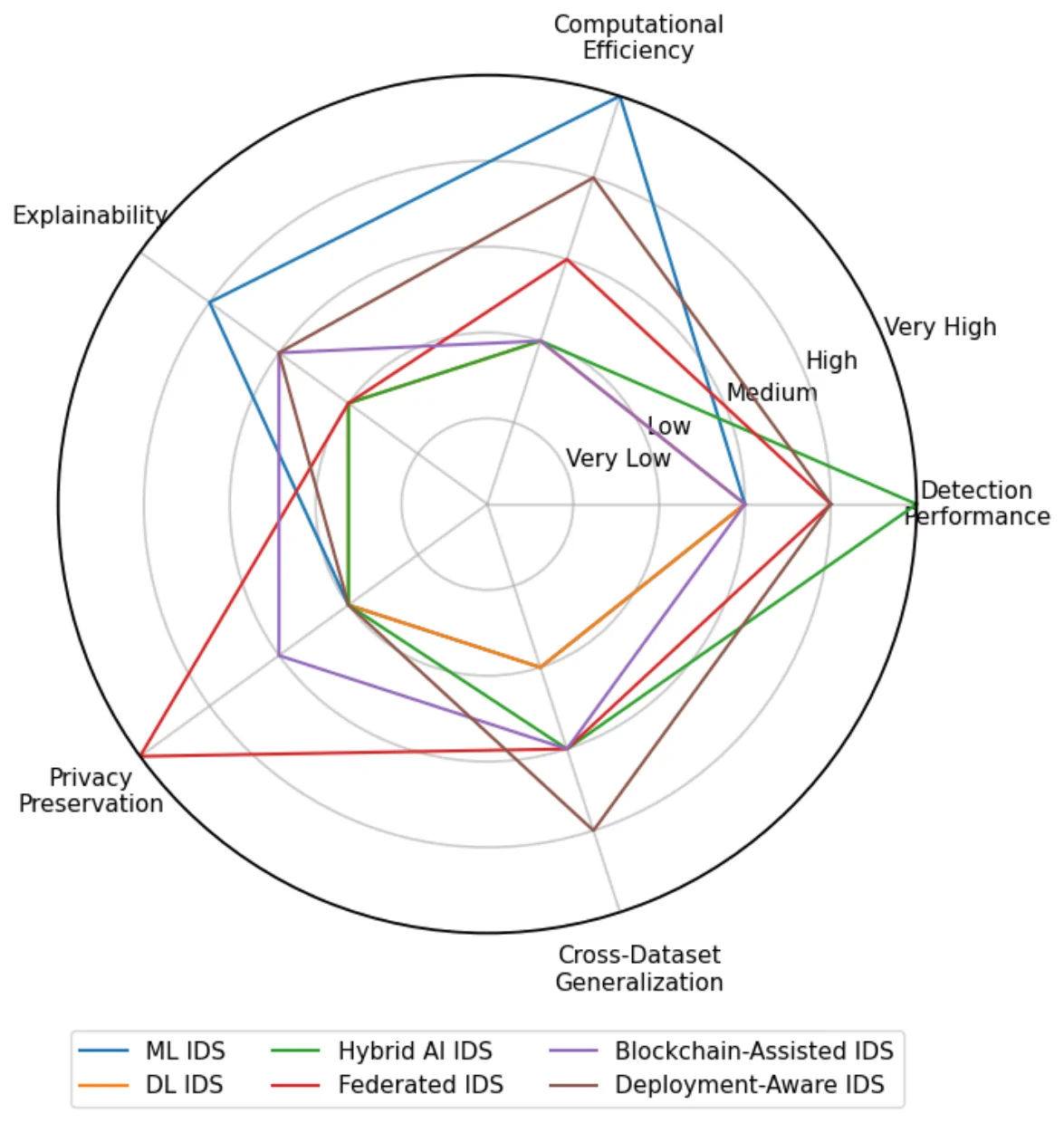
Illustrative qualitative synthesis of the maturity of major AI-powered IDS paradigms across detection performance, explainability, privacy preservation, computational efficiency, and cross-dataset generalization. The figure provides a conceptual visual summary of the reviewed literature and does not represent quantitative measurements or a formal scoring system.

Figure 7 summarizes this transition by illustrating the complementary strengths of successive AI paradigms. As with the previous conceptual comparison, this figure provides an illustrative qualitative synthesis of the recurring patterns identified across the reviewed literature and is intended as a conceptual visual summary rather than a quantitative evaluation or formal scoring system. Rather than identifying a universally superior solution, the reviewed evidence indicates that each paradigm addresses different dimensions of deployment readiness. Traditional machine learning emphasizes efficiency and interpretability, deep learning strengthens feature representation and detection capability, whereas explainable AI, Federated Learning, and blockchain progressively extend IDS functionality toward transparency, collaborative intelligence, privacy preservation, and decentralized trust. Consequently, the maturity of IoMT intrusion detection should be understood as the progressive integration of complementary capabilities rather than continual replacement of existing approaches. Beyond AI-powered intrusion detection itself, deployment-ready IoMT cybersecurity also depends on the security and resilience of the underlying communication infrastructure. IoMT devices increasingly rely on mobile and wireless communication technologies to support continuous patient monitoring, telemedicine, and real-time clinical decision-making. Consequently, secure wireless communication, authenticated network access, and electromagnetic compatibility should be considered complementary deployment requirements alongside AI-powered IDSs. Although these aspects were not a primary focus of the present review, they represent important practical considerations for real-world IoMT deployment and future research toward trustworthy end-to-end Healthcare 5.0 ecosystems.

Perhaps the most important finding emerging from this review is the persistent disconnect between benchmark success and operational deployment. Although many AI-powered IDSs achieve excellent performance under controlled laboratory conditions, comparatively few studies demonstrate robust generalization across heterogeneous healthcare environments or evaluate long-term deployment characteristics. This observation suggests that benchmark accuracy should no longer be regarded as the principal indicator of IDS maturity. Instead, deployment readiness has emerged as the defining measure of practical effectiveness, requiring AI systems to balance predictive capability with robustness, transparency, efficiency, scalability, and clinical usability.

### 4.2. Fundamental Trade-Offs in AI-Powered IoMT Intrusion Detection

The reviewed literature demonstrates that advances in AI-powered IoMT intrusion detection are increasingly governed by a series of interconnected trade-offs rather than improvements in individual performance metrics. As AI models become more sophisticated, gains in one dimension frequently introduce constraints in another, making deployment readiness an optimization problem involving multiple competing objectives rather than a single measure of detection performance.

**Figure 8:**
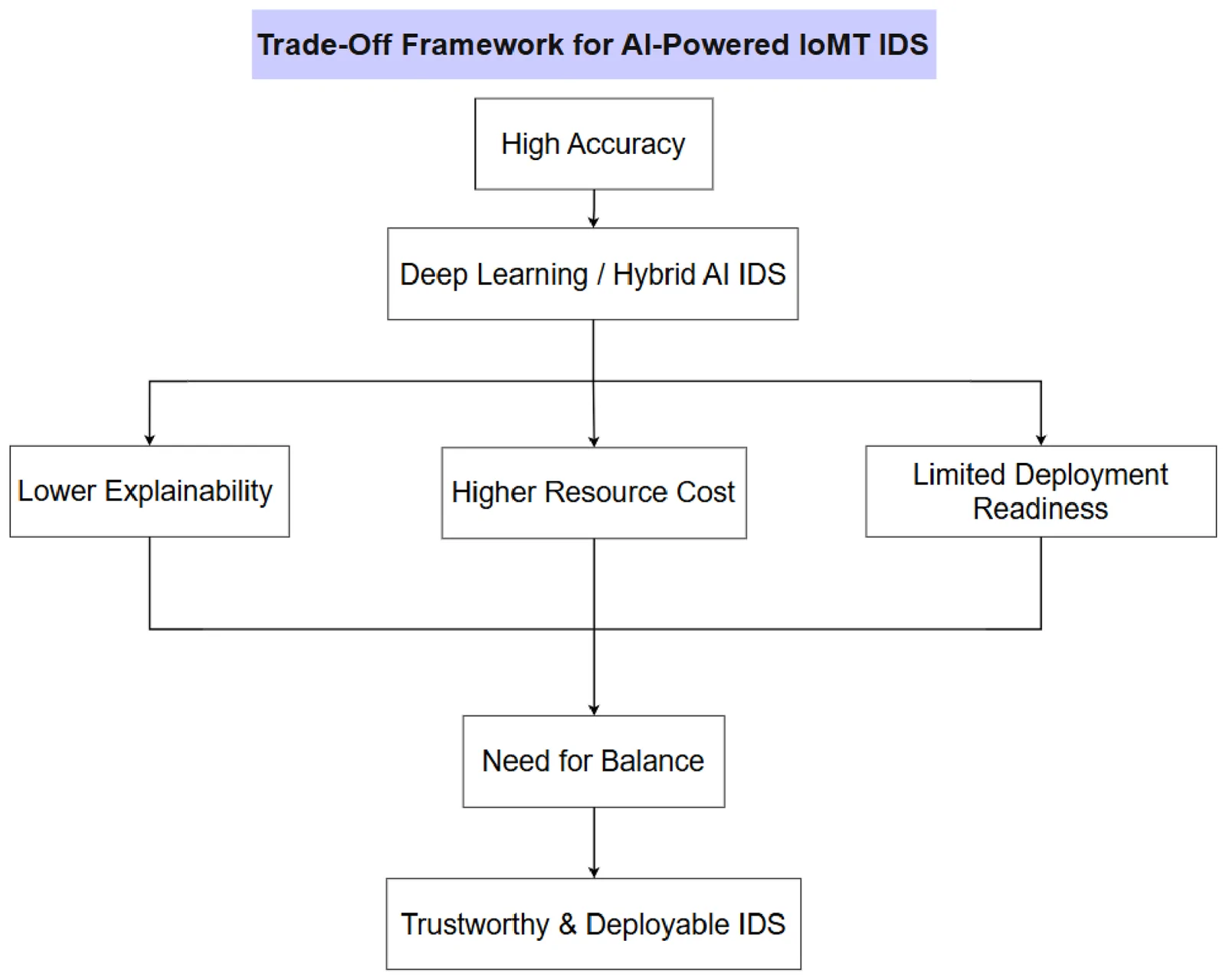
Trade-off framework for AI-powered IoMT intrusion detection systems. Although advanced AI architectures improve detection capability, they simultaneously introduce challenges related to explainability, computational efficiency, privacy, scalability, and deployment.

Figure 8 illustrates these relationships by highlighting that detection capability, explainability, privacy preservation, computational efficiency, scalability, and operational trust should be considered complementary design objectives rather than independent optimization targets.

Three trade-offs consistently emerge across the reviewed literature. First, improvements in predictive capability are frequently accompanied by reduced interpretability, increasing the importance of explainable and trustworthy AI in healthcare environments where transparency and accountability remain essential [2,29,105,106]. Second, collaborative intelligence through Federated Learning and blockchain improves privacy preservation and decentralized trust but introduces communication overhead, heterogeneous data distributions, synchronization complexity, and adversarial risks [31,32,34,44,46,88,124,125]. Third, increasingly sophisticated AI architectures generally require greater computational resources, motivating lightweight AI, TinyML, edge intelligence, and resource-aware model optimization for deployment on constrained IoMT devices [1,29,81,96].

These observations demonstrate that deployment readiness cannot be achieved by maximizing individual performance metrics. Instead, successful IDS architectures must balance detection capability, explainability, privacy preservation, computational efficiency, scalability, and operational trust according to the requirements of specific healthcare environments. These major trade-offs are summarized in Table 14.

### 4.3. Implications for Healthcare 5.0

The findings synthesized throughout this review indicate that IoMT intrusion detection is entering a new stage of maturity in which cybersecurity should be viewed as an integrated healthcare capability rather than an isolated detection function. AI-powered IDSs are increasingly expected to support intelligent clinical services, protect distributed healthcare infrastructures, facilitate secure collaboration across institutions, and provide transparent decision support for clinicians and cybersecurity professionals. Consequently, future healthcare cybersecurity will depend less on identifying a single optimal AI algorithm than on integrating complementary technologies capable of operating reliably within heterogeneous Healthcare 5.0 environments.

This systems-oriented perspective also changes how IDS effectiveness should be evaluated. Rather than emphasizing predictive accuracy alone, deployment-ready systems should demonstrate robust generalization, computational efficiency, explainability, interoperability, resilience, privacy preservation, and long-term operational reliability. Collectively, these characteristics indicate that the future of IoMT intrusion detection lies in system integration rather than algorithmic competition.

Overall, the reviewed evidence demonstrates that AI-powered IoMT intrusion detection has evolved from isolated machine learning models into intelligent, collaborative, and deployment-oriented cybersecurity ecosystems. This conceptual transition provides the foundation for the future research directions presented in the following section.

## 5. Future Research Directions

The evidence synthesized throughout this review indicates that AI-powered intrusion detection for the Internet of Medical Things (IoMT) is entering a new stage of maturity in which future progress will depend less on developing increasingly sophisticated detection algorithms than on engineering intelligent, trustworthy, and deployment-ready cybersecurity ecosystems. The reviewed literature consistently demonstrates that benchmark accuracy alone is no longer sufficient for clinical adoption. Instead, next-generation intrusion detection systems (IDSs) must balance generalization, explainability, privacy preservation, computational efficiency, interoperability, and operational validation to support Healthcare 5.0 services and Remote Patient Monitoring (RPM) infrastructures [1,2,13,15,22,23,24,52,102].

**Figure 9:**
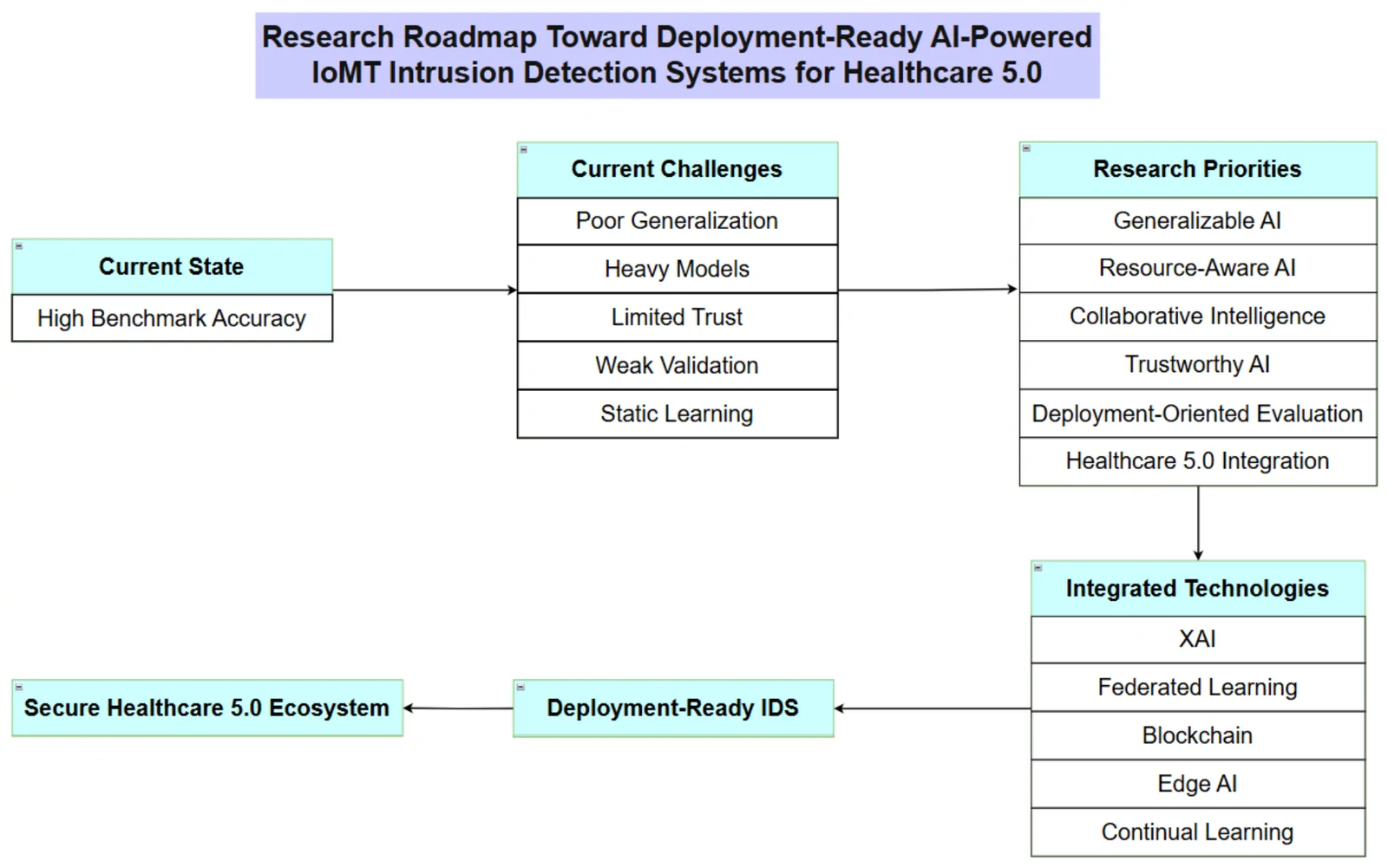
Strategic research roadmap for next-generation AI-powered IoMT intrusion detection systems. Future progress depends on integrating generalizable AI, resource-aware intelligence, collaborative learning, trustworthy AI, deployment-oriented validation, and Healthcare 5.0 integration within unified cybersecurity architectures.

Figure 9 summarizes the strategic research roadmap synthesized from this review. Rather than proposing isolated research directions, the framework highlights complementary priorities that collectively support the development of trustworthy, scalable, resource-efficient, and deployment-ready AI-powered IoMT intrusion detection systems.

### 5.1. Strategic Research Priorities

The reviewed literature identifies five strategic priorities that should guide future research, as summarized in Table 15.

First, improving model generalization remains essential. Future IDSs should move beyond benchmark-specific learning by emphasizing cross-dataset validation, multicentre benchmarking, continual learning, transfer learning, domain adaptation, and foundation models capable of learning generalized behavioural representations rather than dataset-specific attack characteristics [24,40,41,59,135,137,139].

Second, resource-aware intelligence will become increasingly important as AI-powered IDSs are deployed on wearable sensors, implantable devices, edge gateways, and battery-powered RPM platforms. Lightweight deep learning, TinyML, model compression, pruning, knowledge distillation, and edge-native AI should therefore be prioritized to balance predictive capability with latency, memory consumption, and energy efficiency [1,29,81,96,130,131,132,133,134].

Third, collaborative intelligence should evolve beyond privacy-preserving learning toward secure multi-institutional healthcare cybersecurity. Future research should strengthen Federated Learning through personalized aggregation, resilient collaborative optimization, secure aggregation, adaptive cyber threat intelligence, and lightweight blockchain-assisted trust management capable of supporting geographically distributed healthcare infrastructures [31,32,34,44,46,88,115,124,125].

Fourth, trustworthy AI should become an integral design principle rather than a post-deployment enhancement. Explainability, robustness, fairness, governance, accountability, privacy preservation, and meaningful human oversight should be incorporated throughout the IDS lifecycle to support transparent, reliable, and clinically acceptable cybersecurity decision making [2,26,105,106,113,114].

Finally, future evaluation methodologies should extend beyond predictive performance by incorporating standardized deployment-oriented metrics, including cross-dataset robustness, computational efficiency, interoperability, explainability, scalability, resilience, energy consumption, clinical usability, and long-term operational reliability. Such multidimensional evaluation will provide stronger evidence of real-world deployment readiness than conventional accuracy-based benchmarking alone [22,23,24,52].

**Figure 10:**
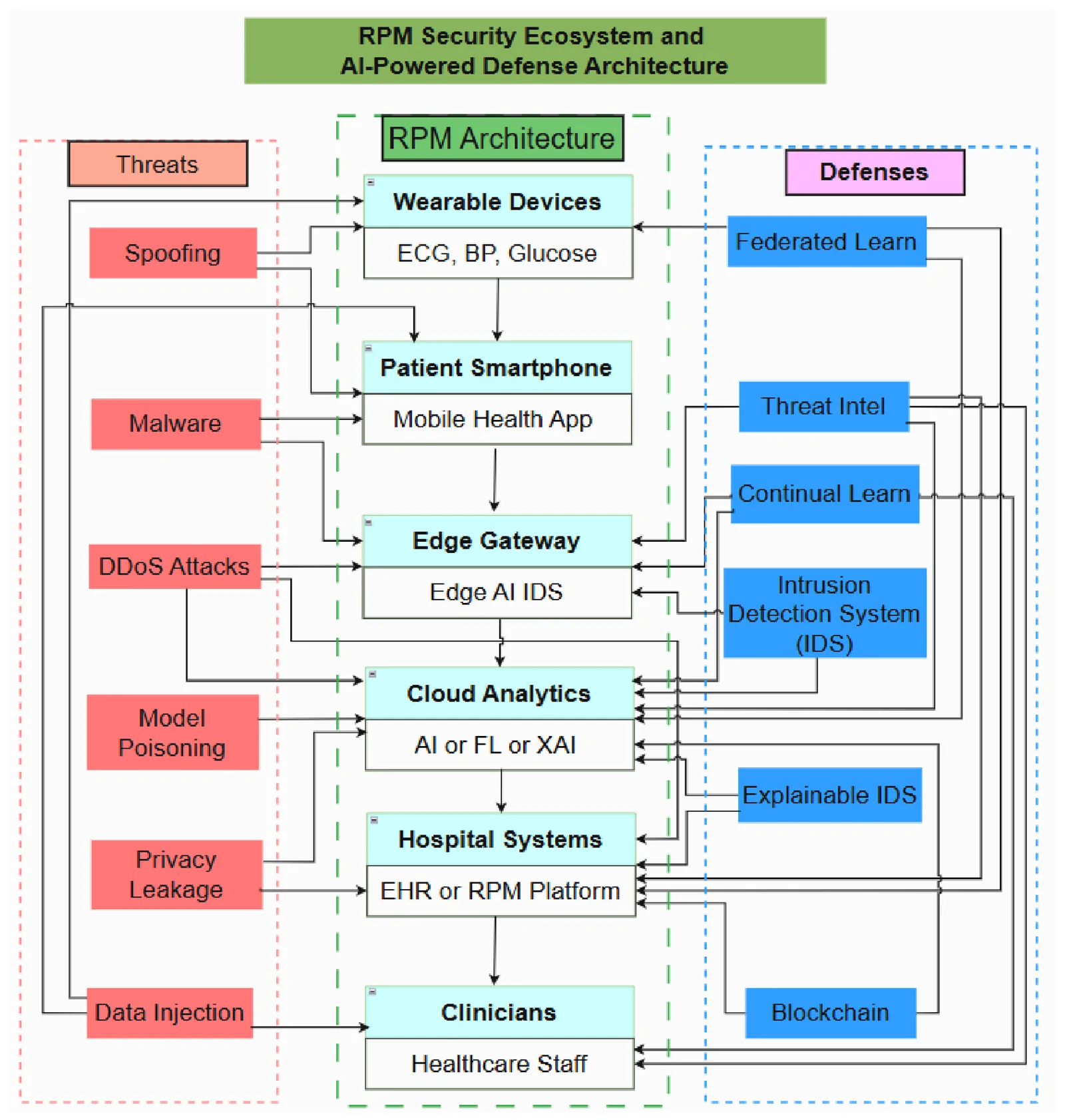
Integrated Healthcare 5.0 cybersecurity ecosystem combining AI-powered intrusion detection, explainable AI, collaborative intelligence, blockchain-assisted trust management, continual learning, and edge-cloud computing for deployment-ready healthcare security.

Figure 10 illustrates how these strategic priorities converge within an integrated Healthcare 5.0 cybersecurity ecosystem. Rather than functioning as independent technologies, AI-powered intrusion detection, Explainable AI, Federated Learning, blockchain-assisted trust management, continual learning, cyber threat intelligence, and edge–cloud computing should operate as complementary components of a unified security architecture capable of protecting increasingly interconnected healthcare environments. The red, green, and blue dashed boxes group cybersecurity threats, RPM architecture components, and defensive technologies, respectively, while the arrows indicate the threat pathways and defensive relationships associated with the individual RPM components.

### 5.2. Strategic Outlook

Collectively, the reviewed evidence indicates that the future of IoMT intrusion detection will be defined by system integration rather than algorithmic competition. Next-generation IDSs should combine generalizable AI, resource-aware intelligence, collaborative learning, trustworthy AI, and deployment-oriented validation within interoperable Healthcare 5.0 cybersecurity ecosystems capable of operating reliably across heterogeneous healthcare infrastructures. Achieving this transition will require closer integration between AI research, cybersecurity engineering, healthcare practice, and regulatory governance to ensure that intelligent intrusion detection systems are not only accurate, but also trustworthy, scalable, resilient, and clinically deployable.

### 5.3. Limitations of This Review

Although this review provides a comprehensive synthesis of recent advances in AI-powered intrusion detection systems for the Internet of Medical Things (IoMT), several limitations should be considered when interpreting the findings.

First, the review was restricted to English-language publications published between 2021 and 2026 and searched across eight major electronic databases. Although this strategy provided broad coverage, relevant studies published outside the selected period, in other languages, or indexed exclusively in alternative databases may not have been captured.

Second, Google Scholar accounted for 4045 of the 5127 records identified (79%) and was searched using relevance-ranked, publication-year intervals rather than the structured database querying used for the other seven sources. This disproportionate contribution introduces a potential selection-bias risk because retrieval from Google Scholar depends partly on ranking and screening position rather than complete database-wide coverage. Consequently, some recent, less-visible, or narrowly indexed studies meeting the eligibility criteria may not have been identified. This risk was partially mitigated by applying the same predefined eligibility criteria, full-text assessment, and methodological quality evaluation to records retrieved from all databases. Nevertheless, the dominance of Google Scholar remains a limitation of the search strategy and should be considered when interpreting the completeness and representativeness of the evidence base.

Third, a quantitative meta-analysis was not performed because the included studies differed substantially in datasets, attack scenarios, AI methodologies, evaluation metrics, and validation protocols. Accordingly, a structured qualitative synthesis was considered more appropriate than statistically combining highly heterogeneous evidence.

Finally, the formal structured synthesis was limited to the 24 primary studies that satisfied all predefined eligibility and quality assessment criteria. Additional supporting studies were incorporated to provide conceptual context, methodological background, Healthcare 5.0 perspectives, and discussion of emerging research directions, but they were not included in the formal comparative analysis.

Despite these limitations, this review provides a transparent and comprehensive synthesis of current AI-powered IoMT intrusion detection research while identifying the principal scientific and operational challenges that must be addressed to achieve trustworthy, deployment-ready healthcare cybersecurity.

## 6. Conclusions

This review synthesized evidence from 24 primary studies, supported by 115 complementary studies published between 2021 and 2026, to critically examine the evolution of Artificial Intelligence (AI)-powered Intrusion Detection Systems (IDSs) for the Internet of Medical Things (IoMT). The findings demonstrate that AI has substantially advanced intrusion detection by enabling intelligent behavioural analysis, adaptive threat detection, and automated cybersecurity decision support. However, the review also shows that improvements in benchmark performance alone are insufficient to support real-world healthcare deployment.

Across the six analytical dimensions examined in this review—detection performance, explainability, privacy preservation, computational efficiency, benchmarking practices, and cross-dataset generalization—the evidence consistently indicates that advances in detection capability have outpaced progress in explainability, privacy-preserving collaboration, computational efficiency, benchmarking consistency, and model generalization. This imbalance helps explain why many AI-powered IDSs achieve excellent benchmark results while remaining insufficiently mature for routine deployment in Healthcare 5.0 environments.

A central finding of this review is that IoMT intrusion detection is undergoing a fundamental transition from algorithm-centred research toward deployment-oriented cybersecurity. Although machine learning, deep learning, hybrid AI, Explainable Artificial Intelligence (XAI), Federated Learning (FL), blockchain-assisted security, and edge intelligence have significantly improved detection capability, their practical value ultimately depends on achieving robust cross-dataset generalization, explainability, privacy preservation, computational efficiency, scalability, interoperability, and long-term operational reliability. Collectively, the evidence indicates that deployment readiness, rather than benchmark accuracy, has become the defining challenge for next-generation AI-powered IoMT cybersecurity.

This review further demonstrates that future progress will depend less on developing increasingly sophisticated detection algorithms than on integrating complementary technologies within unified, trustworthy, and deployment-aware cybersecurity architectures. Explainability, collaborative intelligence, adaptive learning, standardized benchmarking, and rigorous real-world validation should therefore be regarded as essential design requirements rather than optional enhancements for clinically deployable intrusion detection systems.

Overall, this review provides a comprehensive synthesis of the current evidence, identifies the principal barriers limiting operational adoption, and establishes a deployment-oriented framework for future research. By consolidating advances in AI-powered intrusion detection within a unified analytical perspective, it offers researchers, healthcare practitioners, industry stakeholders, and policymakers a practical foundation for developing trustworthy, interoperable, scalable, and clinically deployable cybersecurity systems capable of supporting the next generation of Healthcare 5.0.

## Figures and Tables

**Table 1:** Coverage comparison of analytical dimensions across representative IoMT intrusion detection review papers.

Survey	Yr.	Detect.	XAI	Privacy	Efficiency	Bench.	General.	Deploy.	Key Contribution and Research Gap
Siqueira et al. [23]	2025	✓	✓	△	△	✓	△	✓	Healthcare 5.0 IDS and XAI survey; limited cross-dataset validation and deployment analysis.
Naghib et al. [22]	2025	✓	△	△	△	✓	△	△	Comprehensive IoMT IDS taxonomy; lacks deployment-oriented evaluation framework.
Pinto et al. [31]	2025	△	—	✓	△	△	△	△	Federated learning for IoMT security with emphasis on privacy preservation.
Makris et al. [32]	2025	✓	—	✓	△	△	△	△	Focuses on federated IDS challenges rather than real-world deployment.
Matthew et al. [1]	2025	△	—	△	△	—	—	✓	Healthcare 5.0 overview; IDS evaluation is not the primary focus.
Mulo et al. [29]	2025	✓	✓	△	△	△	—	△	Deep learning for IoMT security; limited benchmarking and deployment discussion.
Trivedi et al. [5]	2025	△	△	△	—	—	—	✓	Remote Patient Monitoring security review emphasizing operational environments.
Myrzashova et al. [34]	2023	—	△	✓	—	—	—	△	Privacy-preserving medical AI using federated learning and blockchain.
Adohinzin et al. [24]	2026	✓	✓	✓	✓	✓	✓	✓	Comprehensive IoMT IDS review; limited integration of Healthcare 5.0 deployment within a unified research roadmap.
**Present Review**	**2026**	**✓**	**✓**	**✓**	**✓**	**✓**	**✓**	**✓**	**Integrates all seven analytical dimensions within a unified deployment-oriented synthesis linking AI detection performance, explainability, privacy preservation, computational efficiency, benchmarking practices, cross-dataset generalization, deployment readiness, Healthcare 5.0, Remote Patient Monitoring, and future research directions.**

Note: The symbols indicate the extent to which each review explicitly addresses the corresponding analytical dimension based on the scope reported in the cited publication. They represent qualitative coverage of review topics and do not constitute quality ratings, methodological superiority, or overall performance rankings. ✓ = explicitly and comprehensively discussed; △ = discussed to a limited extent; — = not explicitly addressed.

**Table 2:** Detailed database search strategy and retrieval records. The literature search covered publications published between 2021 and 2026.

Database	Search Date	Filters Applied	Search Method	Records Retrieved
IEEE Xplore	2–4 June 2026	Publication year; English language; journal articles and conference papers; engineering, cybersecurity, IoT, and AI-related subject areas where available	Database search	244
Google Scholar	2–4 June 2026	Publication year intervals; English-language records; title, abstract, keyword, and source-quality relevance screening	Year-by-year relevance-ranked search	4045
Scopus	5 June 2026	Publication year; English language; article and conference paper document types; computer science, engineering, and medicine-related subject areas	Database search	64
Web of Science	5 June 2026	Publication year; English language; article and proceedings paper document types; computer science, engineering, and healthcare-related categories	Database search	57
ACM Digital Library	5 June 2026	Publication year; English language; research articles and conference proceedings; computing and cybersecurity-related topics	Database search	185
ScienceDirect	6–7 June 2026	Publication year; English language; research articles and review articles; computer science, engineering, and healthcare technology subject areas	Database search	189
SpringerLink	6–7 June 2026	Publication year; English language; journal articles and conference papers; computer science, engineering, and healthcare-related fields	Database search	117
PubMed	6–7 June 2026	Publication year; English language; biomedical, medical informatics, digital health, and healthcare technology relevance	Database search	226
**Total**	—	—	—	**5127**

**Table 3:** Inclusion and exclusion criteria used for study selection.

Category	Inclusion Criteria	Exclusion Criteria
Domain	Studies focusing on IoMT, healthcare cybersecurity, RPM, medical device security, or healthcare network protection	Studies unrelated to healthcare, IoMT, cybersecurity, or digital health technologies
Technology	Studies investigating AI, ML, DL, FL, blockchain, XAI, or intelligent security frameworks	Studies focusing solely on clinical diagnosis or healthcare management without cybersecurity relevance
Security Focus	Studies addressing intrusion detection, anomaly detection, threat intelligence, privacy preservation, authentication, trust management, or secure healthcare communication	Studies unrelated to cyber defence, privacy protection, or attack detection
Research Contribution	Studies reporting IDS architectures, security frameworks, benchmarking analyses, healthcare threat models, or security evaluation methodologies	Studies lacking a clear security contribution or technical evaluation
Publication Type	Peer-reviewed journal articles and conference papers	Editorials, patents, theses, short communications, and non-peer-reviewed publications
Publication Period	Studies published between 2021 and 2026	Publications outside the selected period
Study Quality	Studies providing sufficient methodological or experimental detail	Studies lacking sufficient methodological or technical information
Language	English-language publications	Non-English publications

**Table 4:** Distribution of studies according to quality assessment scores.

Quality Score Range	Count	Interpretation
8–10	20	Strong methodological quality and high relevance to IoMT IDS research
5–7	4	Moderate methodological quality with clear relevance to healthcare cybersecurity
0–4	10	Insufficient methodological rigor or relevance; excluded from the primary synthesis
**Total Assessed Studies**	**34**	Full-text candidate studies evaluated during quality assessment

**Table 5:** Quality assessment scores for the 24 studies included in the primary synthesis.

Study	Q1	Q2	Q3	Q4	Q5	Total	Decision
Siqueira et al. [23] (2025)	2	2	2	2	2	10	Included
Naghib et al. [22] (2025)	2	2	2	2	2	10	Included
Szczepaniuk and Szczepaniuk [35] (2023)	1	2	1	1	2	7	Included
Almotiri et al. [30] (2025)	2	2	2	2	1	9	Included
Islam et al. [43] (2024)	1	2	1	1	1	6	Included
Harshith et al. [44] (2026)	2	2	2	2	1	9	Included
El Khatib et al. [45] (2023)	1	2	1	1	2	7	Included
Ahmed et al. [3] (2024)	2	2	0	2	2	8	Included
Bensaid et al. [46] (2024)	2	2	2	2	2	10	Included
Zubair et al. [47] (2022)	2	2	2	2	1	9	Included
Myrzashova et al. [34] (2023)	2	2	2	1	2	9	Included
Mohammadi et al. [25] (2024)	2	2	2	2	1	9	Included
Akram et al. [48] (2021)	2	2	2	2	2	10	Included
Khurana et al. [49] (2024)	1	2	1	1	1	6	Included
Singhal et al. [2] (2025)	2	2	2	1	2	9	Included
Areia et al. [50] (2024)	2	2	2	1	1	8	Included
Adohinzin and Harrath [24] (2026)	2	2	2	2	2	10	Included
Abreu et al. [51] (2024)	1	2	2	2	1	8	Included
Villafranca et al. [52] (2025)	2	2	2	1	2	9	Included
Trivedi et al. [5] (2025)	2	2	2	2	2	10	Included
Pinto et al. [31] (2025)	2	2	2	1	2	9	Included
Makris et al. [32] (2025)	2	2	2	1	2	9	Included
Matthew et al. [1] (2025)	2	2	2	1	2	9	Included
Mulo et al. [29] (2025)	2	2	2	1	2	9	Included

*Note:* Q1 = relevance to IoMT intrusion detection and healthcare cybersecurity; Q2 = methodological transparency and reproducibility; Q3 = dataset description and evaluation reporting; Q4 = experimental or comparative validation; Q5 = contribution to explainability, privacy preservation, deployment readiness, or broader healthcare cybersecurity. Each criterion was scored from 0 to 2, giving a maximum total score of 10. Studies scoring below 5 were excluded from the primary evidence synthesis.

**Table 6:** Thematic classification of the 24 primary studies included in the review.

Research Theme	Count	Research Focus	Representative Studies
IoMT IDS Surveys and Comprehensive Reviews	3	Comprehensive reviews, taxonomies, benchmarking practices, challenges, and future research directions for IoMT intrusion detection.	Siqueira et al. [23]; Naghib et al. [22]; Adohinzin and Harrath [24]
AI-Powered IDS and Healthcare Threat Detection	6	Application of machine learning and deep learning techniques for attack detection, anomaly detection, threat intelligence, and healthcare cybersecurity.	Almotiri [30]; Mohammadi et al. [25]; Akram et al. [48]; Zubair et al. [47]; Villafranca et al. [52]; Islam et al. [43]
Federated Learning and Privacy-Preserving IDS	5	Collaborative intrusion detection frameworks emphasizing privacy preservation, decentralized learning, secure aggregation, and distributed healthcare security.	Pinto et al. [31]; Makris et al. [32]; Bensaid et al. [46]; Wang et al. [34]; Harshith et al. [44]
Blockchain-Assisted Healthcare Security	4	Blockchain-enabled trust management, auditability, secure data sharing, integrity protection, and decentralized healthcare security.	Szczepaniuk and Szczepaniuk [35]; El Khatib et al. [45]; Myrzashova et al. [34]; Harshith et al. [44]
Healthcare 5.0, IoMT Applications, and Smart Healthcare Technologies	6	Integration of AI, IoMT, Healthcare 5.0, intelligent healthcare infrastructures, smart medical devices, and next-generation healthcare ecosystems.	Matthew et al. [1]; Mulo et al. [29]; Singhal et al. [2]; Islam et al. [43]; Ahmed et al. [3]; Khurana et al. [49]
Remote Patient Monitoring and Healthcare Telemetry Security	2	Security challenges, privacy concerns, threat detection, and protection mechanisms for RPM systems and healthcare telemetry environments.	Trivedi et al. [5]; Matthew et al. [1]
Related AI and Deep Learning Healthcare Security Foundations	3	Supporting studies on AI-driven healthcare security, intelligent monitoring, deep learning applications, and broader cybersecurity foundations.	Abreu et al. [51]; Areia et al. [50]; Mulo et al. [29]

**Table 7:** Detailed characteristics of the 24 primary studies included in the review.

Study	Yr.	Type	Dataset	AI/IDS Method	XAI	FL/BC	Metrics	Key Contribution & Limitation	QS
Siqueira et al. [23]	2025	Survey with XAI case study	WUSTL-EHMS-2020	DT, RF, SVM/SVC, XGBoost	SHAP	—	ACC, PRE, REC, F1	Identifies Healthcare 5.0 IDS gaps and demonstrates XAI benefits; limited public datasets and deployment validation.	10
Naghib et al. [22]	2025	Review	WUSTL-EHMS-2020, ToN-IoT, N-BaIoT, CICIDS2017, Bot-IoT	RF, DT, SVM, KNN, LR, MLP, LSTM, CNN, GAN, DBN	—	Future	ACC, PRE, REC, F1	Comprehensive IoMT IDS taxonomy; limited by inconsistent datasets and weak cross-dataset validation.	10
Szczepaniuk and Szczepaniuk [35]	2023	Blockchain healthcare security framework	Hyperledger Fabric testbed	Blockchain, smart contracts, access control	—	BC	BC validation	Improves blockchain-based trust and auditability; lacks IDS functionality and AI-based attack detection.	7
Almotiri et al. [30]	2025	Experimental malware detection	Ember, BODMAS, PEMachineLearning	LightGBM, XGBoost, DNN, CNN, AE, GAN	—	—	ACC, F1	High malware detection using XGBoost; evaluated on malware datasets rather than IoMT traffic and lacks XAI.	9
Islam et al. [43]	2024	Review paper	No experimental dataset	RF, mRMR, NMIFS, ANOVA, Chi-square, clustering	—	—	Qualitative	Feature selection improves IoMT data processing; lacks IDS evaluation, experiments, and benchmark datasets.	6
Harshith et al. [44]	2026	Federated healthcare architecture	Synthea; UCI Diabetes	FL, DNN, BiLSTM, XGBoost, DP, FedAvg, Blockchain	—	FL+BC	ACC, latency	Privacy-preserving FL healthcare architecture with low latency; validated on disease prediction rather than IDS.	9
El Khatib et al. [45]	2023	Empirical blockchain/IoMT survey	Hospital survey data from Dubai	IoMT, Blockchain, smart contracts, ANOVA, regression	—	BC	ANOVA, regression	Blockchain enhances healthcare trust and performance; lacks IDS implementation and cyberattack detection.	7
Ahmed et al. [3]	2024	Comprehensive IoMT review	Literature synthesis (137 selected studies)	AI, ML, data fusion, blockchain, cryptography, authentication, IoMT security	—	BC	Qualitative	Comprehensive IoMT security review; lacks experimental IDS validation, XAI evaluation, and deployment assessment.	8
Bensaid et al. [46]	2024	Federated blockchain-assisted NIDS	CICIoT2023, Edge-IIoTset	FL, LSTM, Blockchain, SSI, DID, VC, trimmed-mean aggregation	—	FL+BC	ACC, binary/multiclass	Secure blockchain-assisted FL IDS with strong detection; limited by IID assumptions and small-scale validation.	10
Zubair et al. [47]	2022	Experimental healthcare edge IDS	BlueTack Dataset	DNN/MLP, RF, DT, SVM, LR, NB, IF, K-Means, LOF	—	—	ACC, PRE, REC, F1	High Bluetooth-based IoMT IDS performance; limited attack diversity, protocol coverage, and explainability.	9
Myrzashova et al. [34]	2023	Review of BC-based FL	BraTS 2020, Pima, CC-19	BCFL, HE, SMPC, PBFT, secure aggregation	—	FL+BC	Qualitative	BCFL strengthens privacy and trust; limited by latency, computational overhead, and deployment complexity.	9
Mohammadi et al. [25]	2024	Experimental IoMT IDS	CICIoMT2024	CNN, LR, AdaBoost, DNN, RF	—	—	ACC, PRE, REC, F1	High CNN performance on CICIoMT2024; limited explainability and cross-dataset validation.	9
Akram et al. [48]	2021	Trustworthy IDS framework	Healthcare network traffic datasets	ML-based IDS, feature selection, trustworthy IDS framework	Interp.	—	Detection performance	Defines trustworthy IDS principles; effectiveness depends on dataset quality and model optimization.	10
Khurana et al. [49]	2024	Conference review/sourcebook	Various benchmark and in-house datasets	SVM, RF, CNN, LSTM, ensemble learning	Varies	Varies	Varies	Broad review of ML/DL healthcare analytics; not specifically focused on IoMT IDS.	6
Singhal et al. [2]	2025	Multi-domain healthcare AI review	Imaging, SEMG, EDA, EHR, clinical datasets	DL, smart contracts, edge/cloud AI	Conceptual	BC	Qualitative	Demonstrates AI-enabled Healthcare 5.0 integration; limited by deployment cost and latency challenges.	9
Areia et al. [50]	2024	Experimental Dataset & Benchmark	IoMT-TrafficData (Packets & Flows)	DT, RF, SVM, LR, NB, DNN	—	—	ACC, PRE, REC, F1	Introduces a realistic IoMT benchmark dataset with strong ML baselines; lacks XAI, FL, and clinical validation.	8
Adohinzin and Harrath [24]	2026	PRISMA review	CIC-IoMT-2024, WUSTL-EHMS-2020, ECU-IoHT, ToN-IoT, BoT-IoT	ML, DL, Transformers, FL, statistical anomaly detection	SHAP, LIME, Attention	FL	ACC, MCC, cross-dataset	Highlights weak cross-dataset generalization despite high reported accuracy; benchmarking remains inconsistent.	10
Abreu et al. [51]	2024	IoT/vehicular IDS review	UNSW-NB15, KDD Cup 99, NSL-KDD	ML, DL, Hybrid AI IDS	—	—	IDS metrics	AI improves IoT intrusion detection; limited by legacy datasets and weak deployment validation.	8
Villafranca et al. [52]	2025	AI-driven IoT security survey	Edge-IIoTset, BoT-IoT, TON-IoT, MQTTset	AI-driven IDS, DTL, edge intelligence, GenAI, LLM-supported security	Partial	—	Detection/deploy.	Identifies trade-offs among detection, scalability, and explainability; limited real-world deployment evidence.	9
Trivedi et al. [5]	2025	RPM security review	86 research studies; 24 RPM systems	AI anomaly detection, CTI, security protocols, distributed monitoring	Conceptual	—	RPM security	AI improves RPM security resilience; limited by scalability and long-term operational validation.	10
Pinto et al. [31]	2025	Federated learning security review	FL benchmarks and simulated medical IoT environments	FL, asynchronous FL, BC-assisted FL, decentralized anomaly detection	—	FL+BC	Privacy, communication	FL enhances privacy-preserving IDS; limited by communication overhead, poisoning attacks, and scalability.	9
Makris et al. [32]	2025	Federated IDS survey	UNSW-NB15, NSL-KDD, CIC-IDS2017, IIoT benchmarks	FIDS, FL, DRL, asynchronous FL, Byzantine-resilient aggregation	—	FL	Detection, cost	Federated IDS improves privacy and resilience; limited by non-IID data and communication overhead.	9
Matthew et al. [1]	2025	IoMT architecture and edge review	Biomedical telemetry and smart infrastructure benchmarks	Edge intelligence, 5G IoMT, cloud-fog computing, cross-layer fusion	—	—	Architecture	Edge-fog architectures support IoMT monitoring; limited by resource constraints and protocol heterogeneity.	9
Mulo et al. [29]	2025	Deep learning in IoMT review	MESSIDOR, BraTS, physiological signal and healthcare/IoT datasets	CNN, RNN, GAN, AE, DRL, hybrid DL	Conceptual	—	Qualitative	Deep learning enhances IoMT analytics; limited by computational cost, privacy concerns, and edge feasibility.	9

*Note:* ACC = accuracy; PRE = precision; REC = recall; F1 = F1-score; AE = autoencoder; BC = blockchain; BCFL = blockchain-based federated learning; CTI = cyber threat intelligence; DL = deep learning; DP = differential privacy; DRL = deep reinforcement learning; FL = federated learning; FIDS = federated intrusion detection system; HE = homomorphic encryption; IF = isolation forest; Interp. = interpretability; QS = quality score; — = not explicitly addressed.

**Table 8:** Evolution of AI paradigms for IoMT intrusion detection and their deployment characteristics.

AI Paradigm	Why It Emerged	Principal Strength	Remaining Limitation	Deployment Readiness
Traditional Machine Learning	Replace rule-based detection	Lightweight, interpretable, computationally efficient	Handcrafted feature engineering and limited representation learning	Moderate
Deep Learning	Automated feature extraction	High detection capability for complex attack patterns	Computational cost, reduced interpretability, and resource requirements	Moderate–Low
Hybrid and Ensemble AI	Combine complementary learning capabilities	Improved robustness, attack coverage, and predictive stability	Architectural complexity and deployment overhead	Moderate
Adaptive Learning	Address concept drift and evolving attacks	Continuous adaptation to changing threat landscapes	Limited real-world validation and implementation maturity	Emerging

**Table 9:** Comparison of explainability approaches for AI-powered IoMT intrusion detection systems.

Approach	Primary Purpose	Strengths	Remaining Challenges
SHAP	Global and local feature attribution	Provides consistent explanations, improves analyst understanding, and supports forensic investigation	High computational cost for large-scale deep learning models
LIME	Local model interpretation	Model-agnostic and easily integrated with existing IDS architectures	Limited explanation stability across neighbouring instances
Attention Mechanisms	Highlight influential temporal and behavioural patterns	Naturally integrated within deep learning architectures and suitable for sequential traffic analysis	The interpretability of attention weights remains debated
Intrinsically Interpretable Models	Transparent decision-making by design	No post-hoc explanation is required, providing inherently understandable predictions	Often achieve lower predictive performance than highly complex architectures
Explainable Ensemble Models	Balance performance and interpretability	Improve robustness while maintaining explanation capability	Greater implementation complexity and computational overhead

**Table 10:** Core dimensions of trustworthy AI for deployment-ready IoMT intrusion detection systems.

Dimension	Importance for Healthcare IDS	Current Research Challenges
Reliability	Ensures stable intrusion detection under diverse operational conditions	Limited long-term deployment validation and real-world benchmarking
Explainability	Provides transparent and understandable AI-driven security decisions	Computational overhead and inconsistent evaluation methodologies
Robustness	Improves resilience against adversarial attacks and concept drift	Limited research on adversarial robustness in healthcare IoMT environments
Fairness	Promotes consistent performance across heterogeneous devices and clinical settings	Dataset bias, class imbalance, and limited population diversity
Privacy Preservation	Protects sensitive patient information while enabling collaborative learning	Balancing transparency, data utility, and confidentiality
Accountability	Supports governance, auditability, and regulatory compliance	Lack of standardized AI governance and accountability frameworks
Human Oversight	Facilitates clinician and security analyst confidence through human-in-the-loop decision making	Limited human-centered evaluation and usability studies

**Table 11:** Comparison of privacy-preserving and trust-enabling technologies for IoMT intrusion detection systems.

Technology	Primary Objective	Major Advantages	Deployment Challenges	Future Research Direction
Federated Learning	Privacy-preserving collaborative learning	No raw data sharing, regulatory compliance, and collaborative intelligence	Non-IID data, communication overhead, and poisoning attacks	Personalized federated learning, adaptive aggregation, and communication-efficient learning
Blockchain	Decentralized trust and accountability	Immutable audit trails, secure collaboration, and transparent governance	Latency, scalability, interoperability, and resource consumption	Lightweight blockchain and hybrid on/off-chain architectures
Secure Aggregation	Protection of collaborative model updates	Confidential parameter exchange and improved resilience	Cryptographic overhead and implementation complexity	Efficient secure aggregation for resource-constrained IoMT devices
Explainable AI	Transparent decision support	Improved human trust and model interpretability	Computational overhead and lack of standardized evaluation	Clinically meaningful and deployment-oriented explanation frameworks

**Table 12:** Critical deployment barriers and research priorities for real-world AI-powered IoMT intrusion detection systems.

Deployment Barrier	Evidence from the Literature	Impact on Real Healthcare Deployment	Future Research Priority
Cross-Dataset Generalization	Models validated on a single benchmark frequently experience performance degradation when evaluated on unseen healthcare datasets.	Reduced reliability across hospitals, medical devices, and heterogeneous Healthcare 5.0 environments.	Cross-dataset validation, multi-center benchmarking, domain adaptation, transfer learning, and foundation models.
Computational Constraints	State-of-the-art deep learning and hybrid architectures require substantial processing power, memory, and energy resources.	Limited deployment on wearable devices, implantable sensors, and edge gateways.	TinyML, lightweight AI, model compression, pruning, knowledge distillation, and energy-aware inference.
Dynamic Healthcare Environments	Most IDSs rely on static offline learning despite continuously evolving attacks and communication patterns.	Performance deteriorates because of concept drift and emerging cyber threats.	Continual learning, online adaptation, incremental learning, and self-updating IDS frameworks.
Clinical Integration	Most IDSs are evaluated as standalone detection models rather than components of clinical workflows.	Alert fatigue, delayed incident response, and reduced clinical acceptance.	Human-centered AI, clinician-in-the-loop IDS, explainable decision support, and workflow-aware security.
Deployment-Oriented Evaluation	Evaluation primarily emphasizes accuracy, F1-score, and ROC-AUC while overlooking operational characteristics.	Difficulty assessing practical deployment readiness under real-world healthcare conditions.	Standardized evaluation protocols incorporating latency, energy consumption, explainability, robustness, scalability, interoperability, and usability.
Scalability and Interoperability	Limited validation across heterogeneous IoMT ecosystems and multi-institution healthcare infrastructures.	Reduced scalability for regional and national healthcare networks.	Large-scale clinical validation, interoperable IDS frameworks, and standardized healthcare security architectures.

**Table 13:** Deployment-readiness checklist for evaluating AI-powered IoMT intrusion detection systems in real healthcare environments.

Deployment Criterion	Why It Matters	Recommended Evaluation
Latency	Real-time intrusion detection is essential for protecting clinical workflows and Remote Patient Monitoring (RPM) services.	Measure end-to-end inference time, response delay, and throughput under realistic workloads.
Energy Consumption	Battery-powered wearable and implantable medical devices require energy-efficient operation.	Report energy usage (J/inference), battery impact, and edge-device power consumption.
Memory Footprint	Resource-constrained IoMT devices have limited RAM and storage.	Measure RAM utilization, model size, storage requirements, and computational complexity.
Cross-Dataset Generalization	Models should maintain reliable performance across heterogeneous healthcare environments despite differences in medical devices, communication protocols, traffic characteristics, attack distributions, feature engineering, and data preprocessing pipelines.	Report both within-dataset and cross-dataset performance using standardized train-on-one/test-on-another evaluation protocols (e.g., CIC-IoMT-2024 → WUSTL-EHMS-2020, CIC-IoMT-2024 → Edge-IIoTset, or ToN-IoT → MQTT-IoT) to determine whether models learn generalized attack behaviours rather than dataset-specific traffic artefacts.
Explainability	Clinicians and cybersecurity analysts require transparent and interpretable IDS decisions.	Assess SHAP, LIME, attention visualization, feature attribution, and user interpretability studies.
Robustness	Healthcare IDSs must remain reliable under adversarial attacks, noisy data, and concept drift.	Evaluate adversarial robustness, resilience to distribution shift, continual learning, and fault tolerance.
Privacy Preservation	Healthcare data require strong privacy protection during model training and deployment.	Assess Federated Learning, Differential Privacy, Secure Aggregation, Homomorphic Encryption, and data leakage resistance.
Interoperability	Healthcare systems combine heterogeneous medical devices, EHRs, cloud–edge platforms, and communication protocols.	Validate compatibility across multiple IoMT platforms, healthcare standards, and networking protocols.
Usability	IDS outputs should support rapid clinical and security decision making.	Conduct human-centred evaluation, alert quality assessment, workload analysis, and user studies.
Regulatory Readiness	Healthcare AI must comply with legal, ethical, and cybersecurity regulations before deployment.	Assess compliance with healthcare regulations, AI governance frameworks, auditability, and risk management requirements.

**Table 14:** Major trade-offs identified in AI-powered IoMT intrusion detection systems.

Trade-Off	Benefits	Associated Challenges
Performance vs. Explainability	Improved detection accuracy through deep learning and hybrid models	Reduced transparency, limited stakeholder trust, and difficult regulatory acceptance
Privacy vs. Collaboration	Collaborative learning without sharing raw healthcare data	Communication overhead, non-IID data, poisoning attacks, and trust-management challenges
Accuracy vs. Resource Efficiency	Enhanced threat detection capability	Increased computational cost, latency, memory usage, and energy consumption
Transparency vs. Security	Improved interpretability and auditability	Potential information leakage and explanation-guided evasion attacks
Centralization vs. Decentralization	Global visibility and simplified management	Privacy risks, trust concerns, synchronization complexity, and single points of failure

**Table 15:** Strategic research priorities for next-generation AI-powered IoMT intrusion detection systems.

Strategic Priority	Current Challenge	Future Research Direction	Expected Impact
Generalizable AI	Benchmark-specific learning and poor cross-dataset robustness across heterogeneous healthcare environments	Cross-dataset validation, domain adaptation, transfer learning, continual learning, multicentre benchmarking, and foundation models	Reliable and generalizable intrusion detection across diverse Healthcare 5.0 environments
Resource-Aware AI	High computational cost, memory consumption, energy requirements, and limited suitability for resource-constrained IoMT devices	TinyML, lightweight deep learning, model compression, pruning, knowledge distillation, and edge-native intelligence	Practical deployment on wearable devices, implantable sensors, edge gateways, and Remote Patient Monitoring (RPM) platforms
Collaborative Intelligence	Non-IID data, communication overhead, poisoning attacks, trust limitations, and scalability challenges	Personalized Federated Learning, secure aggregation, adaptive collaboration, privacy-preserving optimization, and lightweight blockchain integration	Secure multi-institution collaborative cybersecurity while preserving patient privacy and institutional autonomy
Trustworthy AI	Black-box decision making, limited explainability, governance, fairness, robustness, and human oversight	Explainable AI, inherently interpretable models, trustworthy AI frameworks, AI governance, regulatory compliance, and human-centred IDS design	Improved stakeholder trust, transparent decision support, responsible AI adoption, and greater clinical acceptance
Deployment-Oriented Evaluation	Accuracy-focused benchmarking with limited assessment of operational performance	Standardized deployment metrics incorporating latency, energy consumption, explainability, interoperability, scalability, adversarial robustness, usability, and multicentre validation	Reliable assessment of real-world deployment readiness and operational effectiveness
Healthcare 5.0 Cybersecurity	Fragmented AI solutions and isolated security technologies across distributed healthcare infrastructures	Integrated architectures combining AI-powered IDS, Explainable AI, Federated Learning, blockchain-assisted trust, continual learning, cyber threat intelligence, and edge-cloud intelligence	Intelligent, interoperable, resilient, scalable, and clinically deployable Healthcare 5.0 cybersecurity ecosystems

## Data Availability

No new datasets were generated or analyzed in this review. All literature, datasets, and benchmark resources discussed in this article are available from the original publications and public sources cited in the manuscript.
